# Engineering of the AAV-Compatible Hair Cell-Specific Small-Size Myo15 Promoter for Gene Therapy in the Inner Ear

**DOI:** 10.34133/research.0341

**Published:** 2024-04-25

**Authors:** Shao Wei Hu, Jun Lv, Zijing Wang, Honghai Tang, Hui Wang, Fang Wang, Daqi Wang, Juan Zhang, Longlong Zhang, Qi Cao, Yuxin Chen, Ziwen Gao, Yu Han, Wuqing Wang, Geng-lin Li, Yilai Shu, Huawei Li

**Affiliations:** ^1^ ENT Institute and Department of Otorhinolaryngology, Eye & ENT Hospital, State Key Laboratory of Medical Neurobiology and MOE Frontiers Center for Brain Science, Fudan University, Shanghai, 200031, China.; ^2^ Institute of Biomedical Science, Fudan University, Shanghai, 200032, China.; ^3^ NHC Key Laboratory of Hearing Medicine (Fudan University), Shanghai, 200032, China.

## Abstract

Adeno-associated virus (AAV)-mediated gene therapy is widely applied to treat numerous hereditary diseases in animal models and humans. The specific expression of AAV-delivered transgenes driven by cell type-specific promoters should further increase the safety of gene therapy. However, current methods for screening cell type-specific promoters are labor-intensive and time-consuming. Herein, we designed a “multiple vectors in one AAV” strategy for promoter construction in vivo. Through this strategy, we truncated a native promoter for Myo15 expression in hair cells (HCs) in the inner ear, from 1,611 bp down to 1,157 bp, and further down to 956 bp. Under the control of these 2 promoters, green fluorescent protein packaged in AAV-PHP.eB was exclusively expressed in the HCs. The transcription initiation ability of the 2 promoters was further verified by intein-mediated otoferlin recombination in a dual-AAV therapeutic system. Driven by these 2 promoters, human otoferlin was selectively expressed in HCs, resulting in the restoration of hearing in treated *Otof ^−/−^* mice for at least 52 weeks. In summary, we developed an efficient screening strategy for cell type-specific promoter engineering and created 2 truncated Myo15 promoters that not only restored hereditary deafness in animal models but also show great potential for treating human patients in future.

## Introduction

In the past 2 decades, gene therapy has quickly become a feasible choice for treating many hereditary diseases in human patients, with which normal genetic material or gene editing tools are transferred to cells or tissues to restore their functionalities and therefore the health of human patients [1,2]. Among many delivery tools and technologies, adeno-associated virus (AAV) vectors are prominent viral vehicles that are commonly used for gene therapy due to their clinical safety, high biocompatibility, low pathogenicity, and nonintegrating expression in vivo [3,4]. To date, several AAV-based gene therapy drugs have been approved in Europe and the United States [5–8]. In order to improve the safety and efficacy of gene therapies, we need to maximize the expression of transgenes in the affected cells and minimize off-target expression. However, efficient delivery of gene therapy systems to specific targeted cells via AAVs remains a great challenge [9]. The capsid of AAV can be optimized to enhance cell-type specificity expression of transgenes [3], and currently, a large number of AAV variants have been developed for the specific expression of transgenes with high efficiency in the central nervous system (CNS), liver, retina, and muscle [3,10,11]. However, the existing AAV variants do not meet all research and clinical needs.

In addition to engineering the capsid of AAVs to enhance their cell type tropism, the specific cell-type expression of the transgenes carried by AAV can be achieved at the transcriptional level via gene regulatory elements such as tissue-specific promoters [3,12]. Gene promoters are mainly located in the upstream region of the gene’s start codon, and they control the transcription initiation of the gene by interacting with RNA polymerase [13]. Promoters are thus very important regulatory elements that control the expression range of transgenes in AAV-mediated gene therapy [3]. Most of the available promoters used in gene therapy are ubiquitous promoters, such as the cytomegalovirus immediate-early enhancer/chicken β-actin (CAG) promoter and the cytomegalovirus (CMV) promoter, which support highly efficient transgene expression [14–16]. Ubiquitous promoters have low cell type preference, and they provide long-term and high-level expression of transgenes in multiple types of cells, while ectopic expression of transgenes at a relatively high level may lead to toxic effects in nontargeted cells [16,17]. Furthermore, ubiquitous promoters have some other flaws. For example, the CMV promoter tends to be silenced in the CNS, while the size of the CAG promoter is relatively large (1.7 kb) [18]. Compared with ubiquitous promoters, cell type-specific promoters restrict the ectopic expression of transgenes carried by AAVs, and combining these cell type-specific promoters with particular serotypes of AAVs can further improve the safety of gene therapies [3,19]. In addition to gene therapy, cell type-specific promoters can also be used in tissue regeneration. Accordingly, the manipulation of critical genes that guide the regeneration of a particular cell type from another cell type might be achievable via cell type-specific promoters.

Disabling hearing loss is one of the most common sensory disorders in humans and affects over 5% of the global population [20,21]. To date, 60% of prelingual hearing loss cases can be attributed to genetic causes [22–25], and more than 8,000 variants in more than 150 genes have been implicated in hearing loss [26,27]. The inheritance patterns of genetic hearing loss include autosomal recessive, autosomal dominant, X-linked, and mitochondrial inheritance. Based on the presence of associated phenotypic features, genetic hearing loss can be divided into syndromic and nonsyndromic hearing loss. Approximately 70% of genetic hearing loss cases are nonsyndromic [23], and 80% of nonsyndromic hereditary deafness is inherited in an autosomal recessive manner [23,28]. Cochlear implantation is a very common treatment, but this method cannot treat all cases of hereditary hearing loss and cannot completely restore natural hearing [29,30]. For monogenic sensorineural hearing loss with an autosomal recessive form, hearing defects can be overcome by AAV-mediated gene replacement [14,31,32].

Multiple types of cells perform various biological functions in the inner ear, including hair cells (HCs), supporting cells (SCs), and spiral ganglion cells [18]. Some engineered AAV serotypes prefer HCs, while other serotypes transfect SCs more efficiently. However, none of the naturally occurring AAV serotypes or their variants can specifically target particular cell types in the inner ear with high transfection efficiency [33–35]. HCs play a critical role in sound signal transmission in the inner ear [33,36], and it has been reported that more than 10,000 genes are expressed in these cells [37]. Importantly, many genes are specifically expressed only in HCs and affect hair bundle formation, mechanoreception, and synaptic transmission, including *ATOH1*, *MYO7A*, *MYO15A*, and *OTOF* [38,39]. Considering the importance of HCs and the length of the coding sequence (CDS) in deafness-related genes, it is necessary to engineering a small size HC-specific promoter for gene therapy of hereditary deafness.

As of 2020, more than 200 pathogenic mutations had been identified in the *OTOF* gene, and these were found to be behind 2 to 10% of all cases of hereditary nonsyndromic hearing loss [14,32,40]. The *OTOF* gene is located in the DFNB9 locus (MIM 601071) on chromosome 2p23.3, and it encodes the otoferlin protein that consists of 6 C2 domains and 1 transmembrane domain [41]. In the inner ear, otoferlin is predominantly expressed in HCs and acts as a Ca^2+^ sensor for vesicle exocytosis [36]. Several studies have demonstrated that delivery of *Otof* cDNA via AAVs into HCs is sufficient to restore the hearing of *Otof ^−/−^* mice [14,29,32,42]. However, because the cargo capacity of AAV is about 4.7 kb, while the CDS sequence of *OTOF* is about 6 kb, researchers developed a dual-AAV strategy to deliver the complete *OTOF* cDNA [32,43]. Nowadays, mRNA and protein trans-splicing strategies have been developed to deliver large transgenes via dual or multiple AAVs. Between these 2 strategies, the recombination efficiency of intein-mediated protein splicing has been reported to be higher than *trans*-mRNA splicing [44]. Our previous work demonstrated that an intein-mediated otoferlin protein recombination strategy could completely restore the hearing of *Otof ^−/−^* mice to wild-type (WT) level. In that work, ubiquitous promoters were used to drive the expression of OTOF [41]. Recently, a 1,611-bp inner ear HC-specific Myo15 promoter have been engineered (patent no.: US 2021/0388045 A1). While 1,611-bp HC-specific Myo15 promoter is too large to be used in this strategy, we sought to further reduce this promoter to <1,000 bp.

Here, we developed a “multiple vectors in one AAV” strategy for promoter construction in vivo. We rationally designed 6 truncated-Myo15 promoters, and we screened the HC specificity and the transcription initiation strength of these promoters in the inner ear in vivo using our promoter construction strategy. We identified a 1,157-bp middle-size promoter (mid-Myo15) and further truncated this to a 956-bp mini-Myo15 promoter. These 2 truncated-Myo15 promoters could drive transgene expression specifically in HCs, and the expression of otoferlin driven by these 2 promoters partially restored hearing in *Otof ^−/−^* mice. Our results demonstrate that the truncated-Myo15 promoters had high HC specificity and sufficient transcription initiation strength. Thus, we have developed an in vivo promoter construction strategy for inner ear cells. These truncated-Myo15 promoters and our promoter construction strategy might facilitate AAV-mediated gene therapy for hereditary deafness.

## Results

### Engineering of the Myo15 promoter via the “multiple vectors in one AAV” strategy

**Fig. 1.**
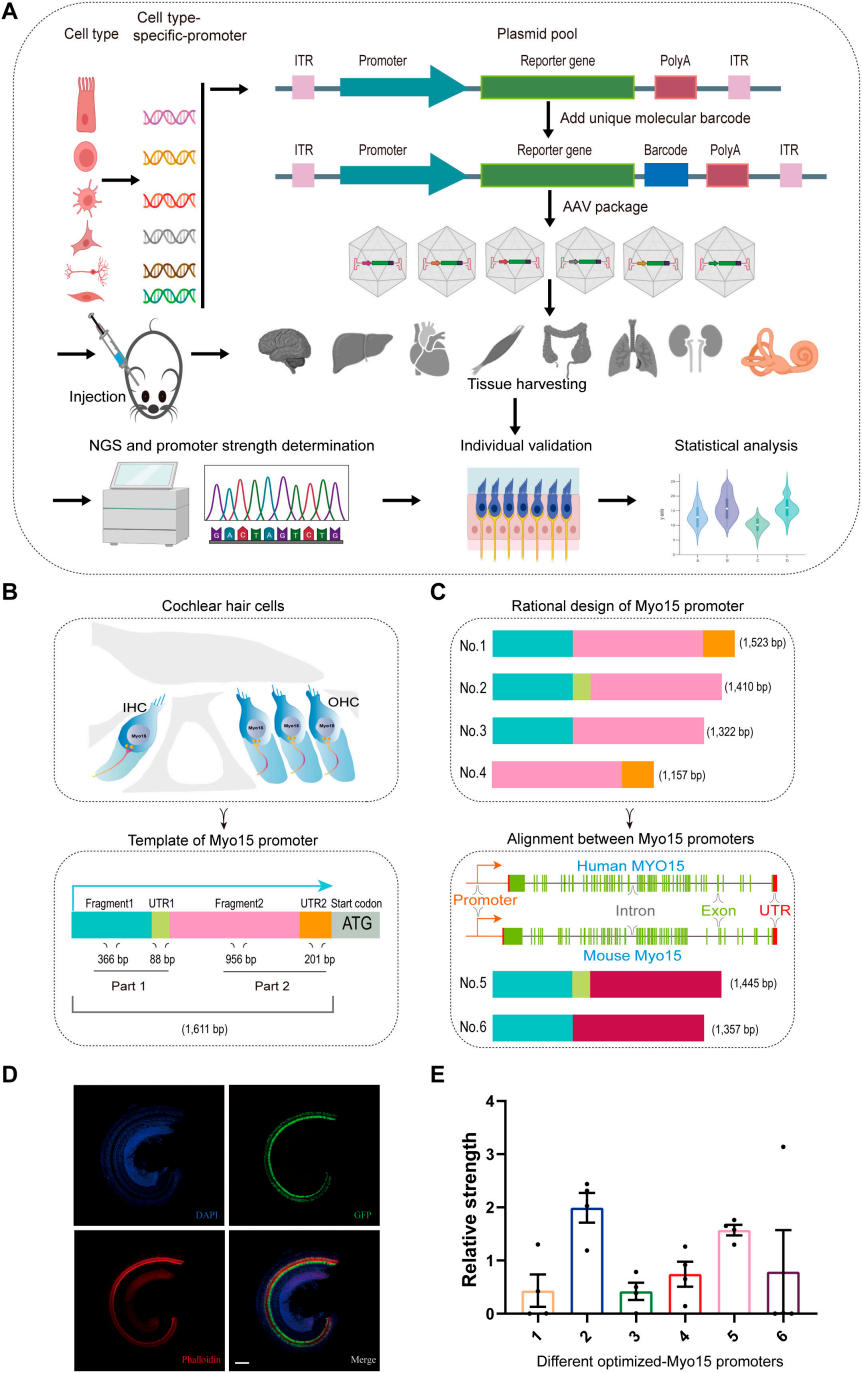
The “multiple vectors in one AAV” cell type-specific promoter construction strategy and the Myo15 promoter truncation strategy. (A) The workflow for the cell type-targeting promoter screening. The cell type-specific promoter sequences were predicted and selected from highly cell type-specific genes, and the promoter sequences were subcloned using an appropriate restriction site combination into pAAV-CMV-GFP, thus replacing the CMV promoter to generate a plasmid pool. Each plasmid was given a unique 15-base molecular barcode, and all plasmids with different molecular barcodes were pooled at equal ratios and packaged into 1 AAV serotype. Next, AAVs were injected into WT mice, and after 3 weeks of expression, the relevant tissues were harvested for sequencing and immunohistochemistry. The transcription initiation strength of each promoter compared to the rest was determined by NGS and the unique molecular barcodes, and promoter strength was ranked based on enrichment of the molecular barcodes in the select tissues, and the top 5% to 10% performers were validated individually. (B) In the inner ear, Myo15 is specifically expressed in HCs. The 1,611-bp Myo15 promoter consists of a 454-bp part 1 and a 1,157-bp part 2. The 454-bp part 1 contains an 88-bp UTR1, and the 1,157-bp part 2 includes a 201-bp UTR2. (C) Design of Myo15 promoters of different sizes. Six different lengths of Myo15 promoters were generated based on the structural components of the Myo15 promoter. The plasmid pool included 6 Myo15 promoters with different sizes that were fused with GFP and corresponding molecular barcodes. These Myo15 promoter plasmids were mixed and packaged into AAV-PHP.eB (4.83 E13 VG/ml), and 1 μl of AAV-PHP.eB (4.83 E10 VG) was injected into the right cochlea of P0 to P2 mice via the RWM. (D) Expression of GFP driven by different lengths of Myo15 promoters. Three weeks after injection, the cochleae were harvested and immunostained to identify the expression of GFP in HCs. (E) The abundance of each molecular barcode was tested via reverse transcription PCR and NGS to evaluate the initial strength of each promoter (*n* = 4). Scale bars: 100 μm. PolyA, polyadenylation site; ITR, inverted terminal repeat.

The promoter is a key component of AAVs that drives transgene expression. Here, we used “multiple vectors in one AAV” strategy to construct cell type-specific promoters in mice. Specifically, promoter sequences of cell type-specific genes were replicated and amplified via polymerase chain reaction (PCR), and these were then subcloned into pAAV-CMV-GFP separately to replace the CMV promoter to generate a plasmid pool. Before packaging into AAV vectors, all plasmids were tagged with a unique 15-bp molecular barcode sequence, whose expression level can be examined via next-generation sequencing (NGS) and linked backed to the specific plasmid [9]. Several weeks after injection, tissues were harvested for immunohistochemistry and sequencing. Promoter strength was ranked based on the enrichment of related barcodes in select tissues (Fig. 1A).

To obtain a small size Myo15 promoter, we established a 2-step truncation strategy. First, we found that that the 1,611-bp Myo15 promoter consists of a 454-bp part 1 and a 1,157-bp part 2. The 454-bp part 1 contains exon 1, and the 1,157-bp part includes exon 2 (Fig. 1B). Exon 1 and exon 2 carry an 88-bp and a 201-bp untranslated region (UTR), respectively (Fig. 1B). We removed the 88-bp UTR in part 1, the 201-bp UTR in part 2, both the 88-bp UTR in part 1 and 201-bp UTR in part 2, and the 454-bp part 1 of the 1,611-bp Myo15 promoter to obtain a 1,523-bp promoter 1, a 1,410-bp promoter 2, a 1,322-bp promoter 3, and a 1,157-bp promoter 4, respectively (Fig. 1C). By comparing the functions of these 4 promoters, we were able to determine whether the 454-bp part 1, 88-bp UTR in part 1, 1,157-bp part 2, or 201-bp UTR in part 2 are necessary to maintain the transcription initiation capacity of the 1,611-bp Myo15 promoter. We also compared and analyzed the sequences of the 1,157-bp part 2 with the predicted human and mouse Myo15 promoter, deleted the phylogenetically unconserved DNA sequence, and generated a 1,445-bp promoter 5 with part 1 and a 1,357-bp promoter 6 in which the 88-bp UTR of promoter 5 was deleted (Fig. 1C). These 2 promoters could further facilitate the engineering of the Myo15 promoter. We constructed 6 plasmids with these 6 different truncated Myo15 promoters and then mixed and packaged these recombinant plasmids into AAV-PHP.eB (Fig. 1A). The AAV-PHP.eB serotype was used in this study because the AAV-PHP.eB serotype exhibit effective inner HCs (IHCs) transduction (almost 100%) [34], and the AAV-PHP.eB serotype mediated gene therapy also performed a higher efficiency in restoring the hearing of *Otof ^−/−^* mice [41,45].

Three weeks after injection of AAV through the round window membrane (RWM) into the right cochlea of WT mice on postnatal days 0 to 2 (P0 to P2), we identified the expression of green fluorescent protein (GFP) in HCs and then evaluated the transcriptional initiation strength of each promoter according to the mRNA abundance of the different molecular barcodes via reverse transcription PCR and NGS. As shown in Fig. 1D, a large amount of GFP was expressed in HCs. Our reverse transcription PCR and NGS results showed that all 6 promoters had sufficient strength to drive the transcription of GFP in HCs (Fig. 1E). According to our design strategy and the length of each promoter, it seemed that the 1,157-bp promoter 4 (which we refer to as the mid-Myo15 promoter) was sufficient to drive the expression of GFP in the cochlea among the 6 different medium-length Myo15 promoters. Although promoter 2 and promoter 5 had relatively higher transcription initiation capacity, the sequence of these 2 promoters was relatively longer. More importantly, the 201-bp UTR in part 2 might not be necessary for the function of the Myo15 promoter. In the second step, we further deleted the particular sequences of the candidate promoter to minimize it according to the first-round results, and we measured their transcription strength and cell-type specificity.

### HC-specific GFP expression driven by the mid-Myo15 promoter

**Fig. 2.**
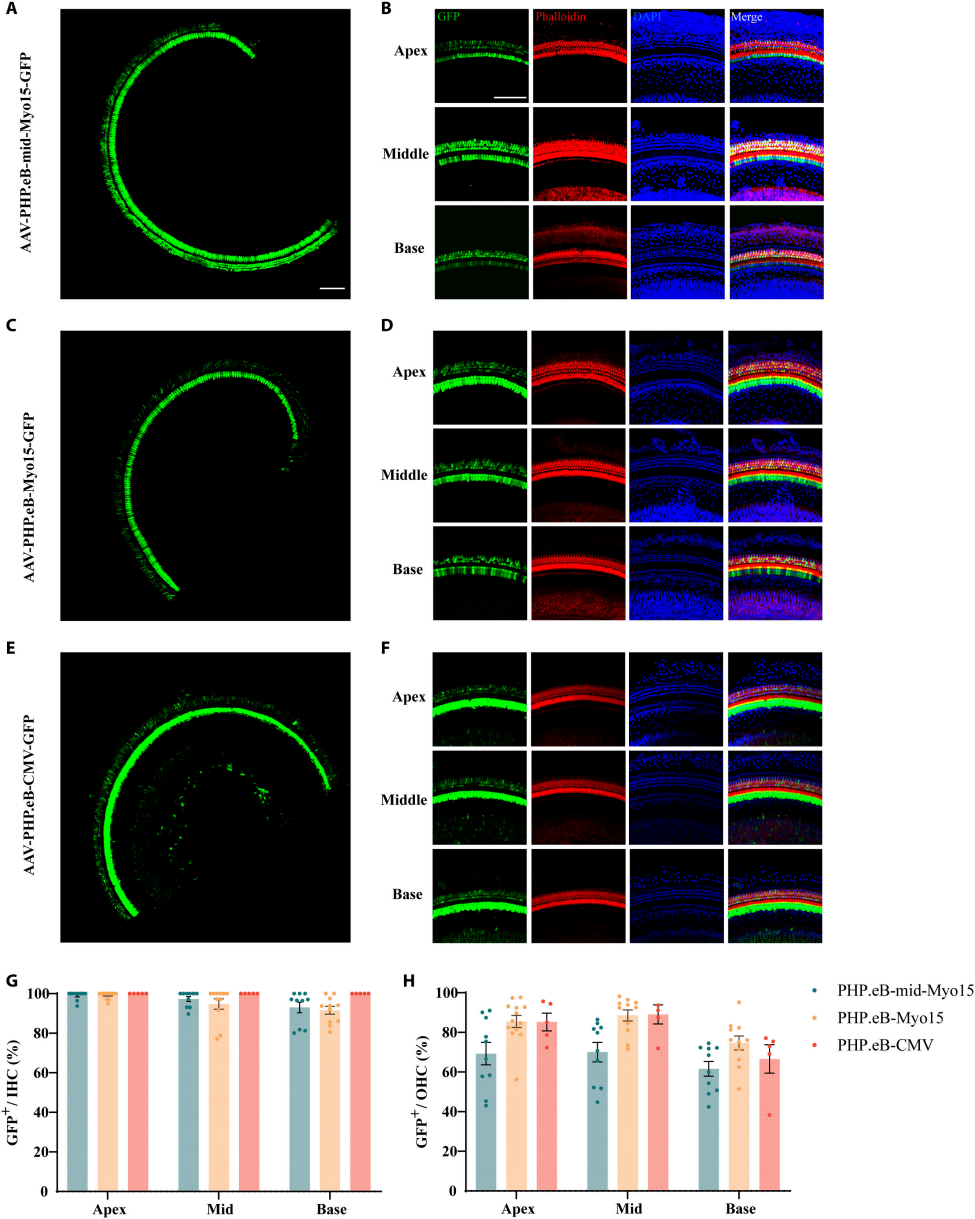
The 1,157-bp mid-Myo15 promoter sufficiently and specifically drove the expression of GFP in HCs. (A to F) GFP distributions in the injected ears after injection of AAV-PHP.eB-mid-Myo15-GFP, AAV-PHP.eB-Myo15-GFP, and AAV-PHP.eB-CMV-GFP, respectively. The GFP distributions in whole basement membranes are shown in the low-magnification views in (A), (C), and (E), respectively. The GFP distributions in the apical, middle, and basal turns are shown in the high-magnification views in (B), (D), and (F), respectively. (G and H) GFP expression efficiency in HCs of the injected ear. The percentages of IHCs and OHCs with GFP expression were determined in the injected ears after injection of AAV-PHP.eB-mid-Myo15-promoter-GFP (*n* = 11 to 13), AAV-PHP.eB-Myo15-GFP (*n* = 10), and AAV-PHP.eB-CMV-GFP (*n* = 5), respectively. Scale bars: 100 μm.

In order to measure the transcription initiation strength of the 1,157-bp mid-Myo15 promoter, we packaged it in AAV-PHP.eB to drive the expression of GFP. The GFP expression efficiency of AAV-PHP.eB-mid-Myo15-promoter-GFP was measured and compared with AAV-PHP.eB-Myo15-GFP (1,611-bp Myo15 promoter [45]) and AAV-PHP.eB-CMV-GFP in mice under otherwise identical conditions. The right cochleae of WT P0 to P2 mice were injected with 1 μl of AAV at a titer of 1E 13 via the RWM. Two weeks after injection, the cochleae were harvested and immunostained. 4′,6-Diamidino-2-phenylindole (DAPI) and phalloidin were used to label the cell nucleus and cochlea HCs, respectively (Fig. 2A to F). IHCs and outer HCs (OHCs) with GFP signal were counted. As shown in Fig. 2G, GFP driven by the mid-Myo15 promoter was expressed in more than 90% of IHCs in the apical, middle, and basal regions of the injected ears, and this was similar to the GFP expression driven by the 1,611-bp Myo15 and CMV promoters. We also observed the specific expression of GFP in OHCs. As shown in Fig. 2H, 2 weeks after injection of AAV, GFP was expressed in about 85%, 89%, and 67% of OHCs in the apical, middle, and basal regions of the injected ears in the AAV-PHP.eB-CMV-GFP group. In the AAV-PHP.eB-mid-Myo15-GFP group, however, GFP expression was reduced to about 69%, 70%, and 62% of OHCs in the apical, middle, and basal regions of the injected ears. Importantly, in the AAV-PHP.eB-Myo15-GFP group, GFP expression was enhanced, in about 86%, 89%, and 75% of OHCs in the apical, middle, and basal regions of the injected ears, comparable to the AAV-PHP.eB-CMV-GFP group. These results clearly demonstrated that the efficiency of the truncate mid-Myo15 promoter reaches the similar level as the native Myo15 promoter and ubiquitous CMV promoter in the IHCs.

It has been reported that administration of transgenes carried by AAV-PHP.eB in the injected ear can transfect the contralateral ear [41]. Therefore, we counted the GFP-positive IHCs in the contralateral ear. As shown in Fig. S1A to D, about 83%, 39%, and 27% of IHCs in the apical, middle, and basal regions of the contralateral ear in the AAV-PHP.eB-mid-Myo15-GFP group expressed GFP. For the OHCs of the contralateral ear, GFP was expressed in about 25%, 13%, and 10% of the OHCs in the apical, middle, and basal regions in the AAV-PHP.eB-mid-Myo15-GFP group, which is lower than AAV-PHP.eB-Myo15-GFP group and AAV-PHP.eB-CMV-GFP group (Fig. S1E). These results combined with the expression range of GFP in IHCs demonstrated that the strength of mid-Myo15 promoter was greater in IHCs compared to OHCs, and the expression of GFP driven by mid-Myo15 promoter was more IHC-specific compared to OHCs. More importantly, the strength of the 1,157-bp mid-Myo15 promoter was strong enough to drive the expression of transgenes in inner ear HCs.

### Restoration of hearing mediated by the mid-Myo15 promoter in DFNB9 mice

Although the cargo capacity of AAV is about 4.7 kb, it has been reported that using an AAV “overloading” strategy to deliver large transgenes can also result in detectable expression of these genes [46,47]. For example, AAVs overloaded with the ∼6.0-kb full-length *Otof* CDS expressed otoferlin in ∼30% IHCs and partially restored the hearing of *Otof ^−/−^* mice [29]. Considering that the length of the mid-Myo15 promoter was only 157 bp longer than our target of 1,000 bp, we wanted to test the transcription initiation capacity of the mid-Myo15 promoter in driving the expression of a therapeutic protein. We designed a dual-AAV approach to deliver human otoferlin protein into the HCs of *Otof ^−/−^* mice using an intein-mediated protein recombination strategy as described above to specifically control the expression of full-length human otoferlin in IHCs by the truncated mid-Myo15 promoter. We packaged AAV-PHP.eB-mid-Myo15-hOtof5-N-S2-Rma-N-intein (AAV-PHP.eB-hOTOF NT, 4,599 bp) and AAV-PHP.eB-mid-Myo15-Rma-C-intein-hOtof5-C-S2 (AAV-PHP.eB-hOTOF CT, 4,853 bp) vectors and injected 1 μl of a 1:1 mix of NT and CT AAVs into the right ears of *Otof ^−/−^* mice at P0 to P2 via the RWM. After the injection of AAVs, we identified the expression of otoferlin protein in IHCs and measured the auditory brainstem response (ABR) in WT, uninjected *Otof ^−/−^* mice, and *Otof ^−/−^* mice injected with dual-AAV that was packaged with the mid-Myo15 promoter. We then evaluated the expression of human otoferlin in *Otof* ^−/−^ mice by dissecting and immunostaining the organs of Corti with a primary antibody against otoferlin at P21 to P30.

**Fig. 3.**
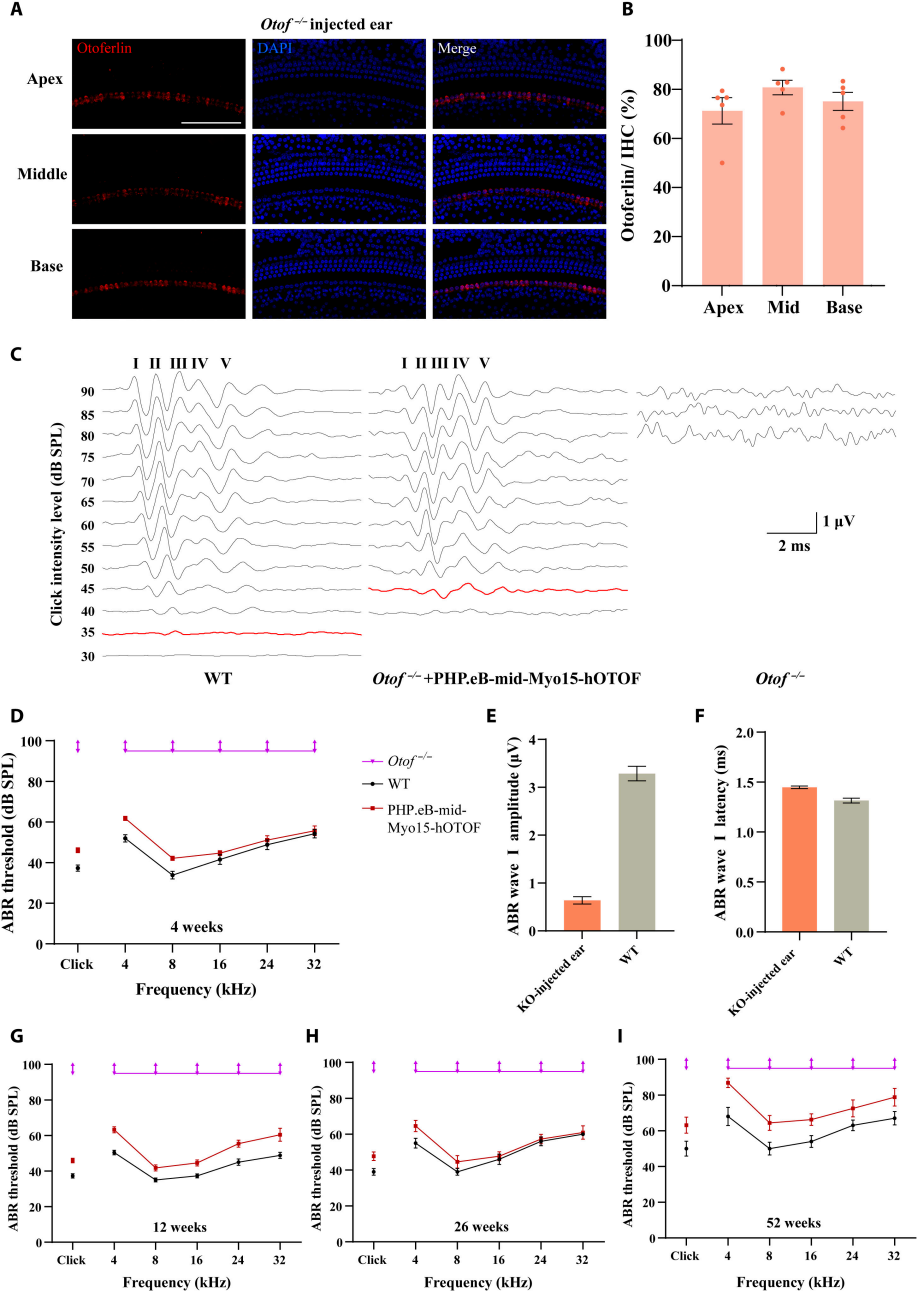
Expression of otoferlin in HCs controlled by the mid-Myo15 promoter restored hearing in *Otof ^−/−^* mice. (A) Expression of otoferlin in IHCs of the inner ear after AAV injection. Otoferlin was expressed in the IHCs of *Otof ^−/−^* mice, including the apical, middle, and basal turns. (B) The number of IHCs with otoferlin was counted in *Otof ^−/−^* mice after treatment. (C) Representative ABR traces in response to broadband click sound stimuli were recorded 4 weeks after treatment. (D) The ABR thresholds in *Otof ^−/−^* mice were recorded for click sound stimuli and pure-tone stimuli 4 weeks after therapeutic injection (WT, *n* = 13, injected mice, *n* = 14; *Otof ^−/−^*, *n* = 8), respectively. (E and F) The ABR wave I amplitude and latency in *Otof ^−/−^* mice were recorded at the 90-dB click sound intensities 4 weeks after therapeutic injection (WT, *n* = 15; injected, *n* = 17), respectively. (G to I) The ABR thresholds in *Otof ^−/−^* mice were recorded for click sound stimuli and pure-tone stimuli 12 weeks (G) (WT, *n* = 13, injected mice, *n* = 11; *Otof ^−/−^*, *n* = 8), 26 weeks (H) (WT, *n* = 5, injected mice, *n* = 11; *Otof ^−/−^*, *n* = 8), and 52 weeks (I) after therapeutic injection (WT, *n* = 5, injected mice, *n* = 8; *Otof ^−/−^*, *n* = 6), respectively. Scale bars: 100 μm. SPL, sound pressure level; KO, knock out.

First, we tested the expression of otoferlin in WT and *Otof ^−/−^* mice. As shown in Fig. S2, otoferlin was expressed in all IHCs in WT mice, while no otoferlin was expressed in IHCs of *Otof ^−/−^* mice. Four weeks after injection of dual-AAV packaged with the mid-Myo15 promoter and human *OTOF* CDS, otoferlin protein was expressed in more than 70% of the IHCs in the injected ears of *Otof ^−/−^* mice, and the localization of otoferlin was consistent with that of WT mice (Fig. S2 and Fig. 3A and B). We also tested the auditory function of *Otof ^−/−^* mice after AAV injection. As shown in Fig. 3C, after 4 weeks injection of dual-AAVs, ABR waves I to V were clearly identifiable, while no identifiable ABR waveforms were elicited in *Otof ^−/−^* mice even at the 90-dB maximum sound pressure level. Compared to the *Otof ^−/−^* mice, the click ABR thresholds in the injected ear were restored to 46.07 ± 1.19 dB, which was about 10 dB higher than the WT level (Fig. 3D). For the tone-burst stimuli, the average thresholds in the mid-Myo15 group were comparable to those of WT mice at 16, 24, and 32 kHz [*P* > 0.05, 2-way analysis of variance (ANOVA)] and approximately 10 dB higher than WT at 4 and 8 kHz (*P* < 0.01) (Fig. 3D). In addition, we analyzed the ABR wave I amplitude and latency of the injected ears in *Otof ^−/−^* mice. The amplitude, which represents the electrical responses of primary auditory neurons to the sound stimuli, was substantially lower than WT (click stimulus at 90 dB; WT, 3.29 ± 0.15 μV; injected ears, 0.64 ± 0.08 μV; *P* < 0.01) (Fig. 3E), while the latency was largely restored to WT levels (click stimulus at 90 dB; WT, 1.32 ± 0.02 ms; injected ears, 1.45 ± 0.01 m; *P* < 0.01) (Fig. 3F). More importantly, dual-AAVs administration markedly improved the auditory function of *Otof ^−/−^* mice for at least 52 weeks in injected ears (Fig. 3G to I). All of these results indicate that the mid-Myo15 promoter is strong enough to drive the expression of therapeutic proteins, like otoferlin, to restore auditory function in cases of hereditary deafness. However, the length of the mid-Myo15 promoter (1,157 bp) was larger than our target (1,000 bp).

### Engineering and verification of the HC-specific mini-myo15 promoter

**Fig. 4.**
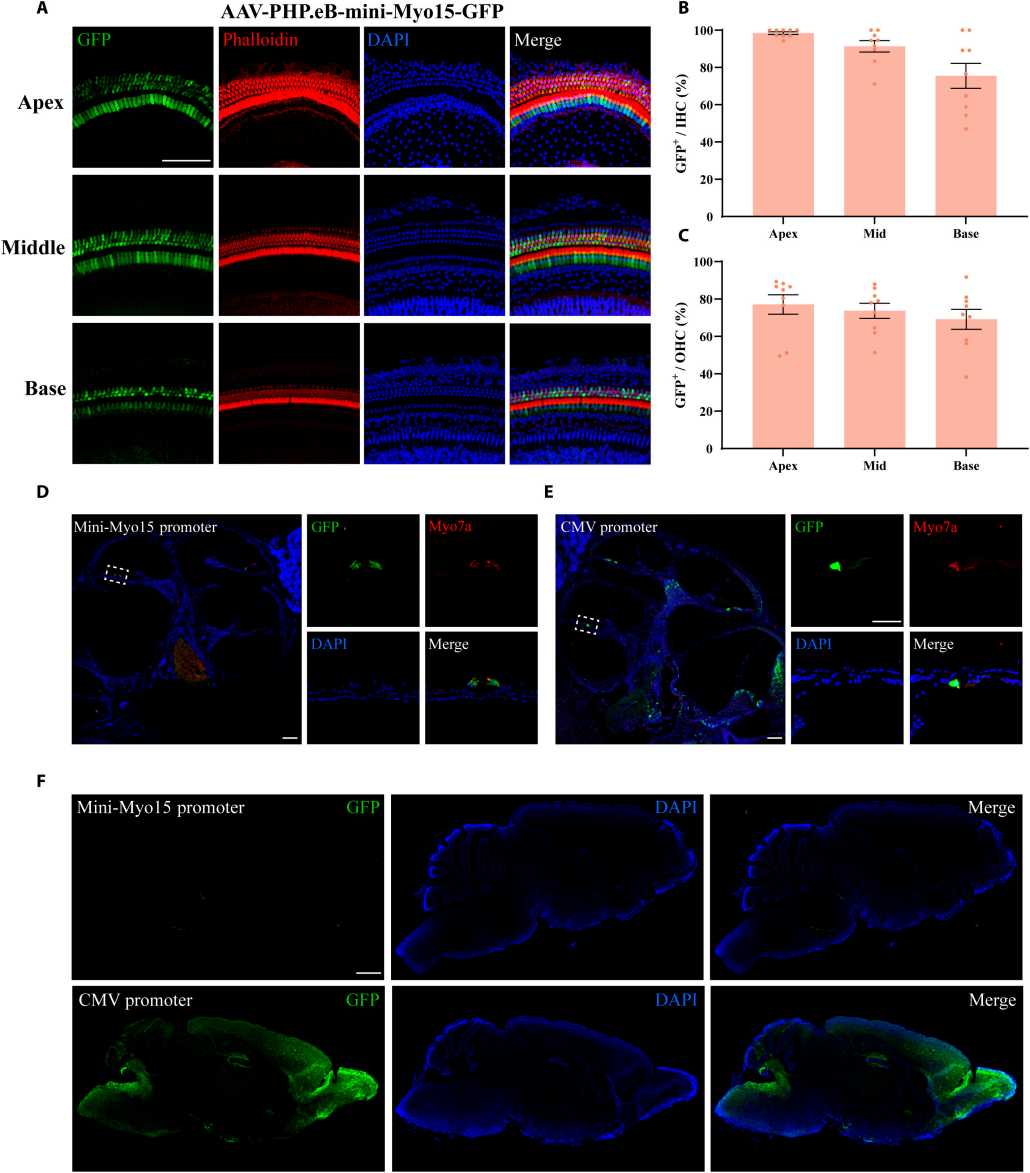
Exclusive expression of transgenes in HCs driven by the mini-Myo15 promoter. (A) Driven by the mini-Myo15 promoter, GFP was specifically expressed in IHCs at the apical, middle, and basal turns. (B and C) GFP expression efficiencies in the apical, middle, and basal turns in IHCs and OHCs, respectively, after injection of AAV-PHP.eB-mini-Myo15-promoter-GFP (*n* = 9). Scale bars: 100 μm. (D and E) Biodistribution of GFP in the cochlea after injection of AAV-PHP.eB-CMV-GFP and AAV-PHP.eB-mini-Myo15-GFP, respectively. Left panel: The overall cochlear GFP distribution in the cochlea. Right panels: Enlarged images of the HC region. Scale bars: 100 μm. (F) Biodistribution of GFP in the brain after administration of AAV-PHP.eB-mini-Myo15-GFP and AAV-PHP.eB-CMV-GFP. Scale bars: 1 mm.

To further increase the length of the transgene packaged by the AAV vector, we tried to truncate a smaller Myo15 promoter. We compared and analyzed the sequences between the 1,611-bp promoter, the 1,157-bp promoter, and the predicted human Myo15 promoter. The 1,611-bp promoter contains exon 1 and exon 2, whereas the 1,157-bp promoter only includes exon 2, and exon 1 and exon 2 contain an 88- and 201-bp UTR, respectively. First, according to the first-round engineering results, we hypothesized that removing the 201-bp UTR of the mid-Myo15 promoter would not affect its strength. We deleted the 201-bp UTR of the mid-Myo15 promoter and obtained a 956-bp mini-Myo15-1 promoter. Second, we designed another mini-Myo15-2 promoter by exchanging the 201-bp UTR with the 88-bp UTR. We separately packaged these 2 promoters into AAV-PHP.eB (titer: 1E13 vector genomes [VG]/ml) to drive the expression of GFP. Two weeks after injection of AAVs (1 μl), we tested the expression pattern of GFP in HCs. As shown in Fig. 4A, the 956-bp mini-Myo15 promoter initiated the expression of GFP in HCs, while the 1,044-bp mini-Myo15 promoter-2 was not functional (Fig. S3). In the AAV-PHP.eB-mini-Myo15-GFP group, about 98%, 91%, and 76% of the IHCs in the apical, middle, and basal regions of the injected ear expressed GFP, while about 77%, 74%, and 69% of the OHCs in the apical, middle, and basal regions in the injected ear were labeled by GFP (Fig. 4A to C). For the contralateral ear, GFP was expressed in about 92%, 32%, and 16% of the IHCs and was expressed in 59 %, 32%, and 18% of the OHCs (Fig. S4). In contrast, in the AAV-PHP.eB-mini-Myo15-2-GFP group neither the IHCs nor the OHCs in the injected ear were labeled by GFP (Fig. S3). All of these results suggest that the 956-bp mini-Myo15 promoter controls the HC-specific expression of transgenes.

### The tissue specificity of the mini-Myo15 promoter

In order to confirm the HC specificity of the mini-Myo15 promoter, we compared the expression pattern of GFP between the AAV-PHP.eB-mini-Myo15-GFP group and the AAV-PHP.eB-CMV-GFP group in the inner ear by cryosectioning of the cochlea 2 weeks after the injection of vectors via the RWM. As shown in Fig. 4D and E, in the AAV-PHP.eB-mini-Myo15-GFP group, GFP was exclusively expressed in HCs (colabeled by phalloidin), while the AAV-PHP.eB-CMV-GFP group showed a broad infection pattern and GFP was expressed in various cell types, including HCs, the lateral wall, the spiral limbus, the spiral ganglion, and the stria vascularis. In order to further confirm the low off-target effect of the mini-Myo15 promoter, we compared the expression pattern of GFP between the AAV-PHP.eB-mini-Myo15-GFP group and AAV-PHP.eB-CMV-GFP group in the CNS 2 weeks after RWM injection of AAV. As shown in Fig. 4F, under the control of the mini-Myo15 promoter, GFP was only rarely expressed in the CNS. In contrast, RWM administration of AAV-PHP.eB-CMV-GFP transduced the cells in the CNS. Under the control of the ubiquitous CMV promoter, GFP was expressed in many brain regions, including the olfactory bulb, the cerebral cortex, the hippocampus, and the cerebellar regions. These results indicate that although AAV-PHP.eB transduced different kind of cells in the inner ear and CNS, the HC-specific mini-Myo15 promoter diminished or inhibited the expression of transgenes in these regions and restricted the expression of transgenes in HCs, which improved the accuracy and safety of gene therapy at the transcriptional level.

### Mini-myo15-promoter-mediated gene therapy rescued the hearing of *Otof* ^−/−^ mice

**Fig. 5.**
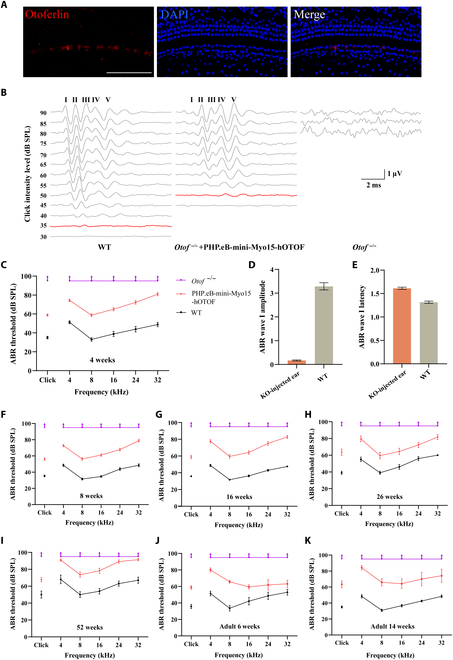
Improvement of auditory function in *Otof ^−/−^* mice after administration of AAV-PHP.eB-mini-Myo15-hOTOF. (A) The expression of otoferlin was restored after injection of AAV-PHP.eB-mini-Myo15-promoter-OTOF in P0 to P2 mice (1 μl). Scale bars: 100 μm. (B) Representative ABR traces in response to broadband click sound stimuli were recorded 4 weeks after AAV administration at P0 to P2. (C) The ABR thresholds of *Otof ^−/−^* mice for click sound stimuli and pure-tone stimuli were recorded 4 weeks after therapeutic AAV injection on P0 to P2 (WT, *n* = 13; injected mice, *n* = 46; *Otof ^−/−^*, *n* = 8). (D and E) The ABR wave I amplitude and the ABR wave I latency in *Otof ^−/−^* mice were recorded at 90-dB click sound intensities 4 weeks after therapeutic injection on P0 to P2 (WT, *n* = 13; injected mice, *n* = 46; *Otof ^−/−^*, *n* = 8). (F to I) The ABR thresholds of *Otof ^−/−^* mice for click sound stimuli and pure-tone stimuli were recorded 2, 4, 6, and 12 months after therapeutic injection (a 1-μl dose) on P0 to P2 (WT, *n* = 13; injected mice, *n* = 44; *Otof ^−/−^*, *n* = 8). (J and K) The ABR thresholds of *Otof ^−/−^* mice for click sound stimuli and pure-tone stimuli were recorded at 2 and 10 weeks after therapeutic injection (1 μl) on P28 (WT, *n* = 5; injected mice, *n* = 11; *Otof ^−/−^*, *n* = 8).

To further test the transcription initiation strength of the mini-Myo15 promoter in driving the expression of therapeutic proteins, we packaged AAV-PHP.eB-mini-Myo15-hOtof5-N-S2-Rma-N-intein (N-fragment AAV, NT) and AAV-PHP.eB-mini-Myo15-Rma-C-intein-hOtof5-C-S2 (C-fragment AAV, CT) vectors and injected 0.5 μl of NT AAV or 1 μl of a 1:1 mix of NT and CT AAVs into the right ears of *Otof ^−/−^* mice at P0 to P2 via the RWM. Four weeks after injection, otoferlin protein was successfully recombined expressed in HCs in *Otof ^−/−^* mice (Fig. 5A). This result demonstrated that the mini-Myo15 promoter possessed sufficient strength to drive the expression of functional proteins in vivo. We also evaluated the safety of the mini-Myo15-promoter-driven therapeutic system. A total of 1 μl of a 1:1 mix of N- and C-fragment therapeutic AAV was injected into WT mice at P0 to P2. The ABR was assessed 12 weeks after injection, and no significant differences were observed between the injected ear and noninjected ear of the mice at any frequency (Fig. S5A), suggesting that the mini-Myo15-promoter-driven therapeutic system was well tolerated.

In order to test whether the recombination of exogenous full-length human otoferlin protein could restore the hearing of *Otof ^−/−^* mice, we measured ABR thresholds in injected *Otof ^−/−^* mice. As shown in Fig. 5B to E, at 4 weeks after injection of dual AAVs packaged with the mini-Myo15 promoter and human *OTOF* CDS, the hearing of *Otof ^−/−^* mice was markedly restored in the injected ear, while the hearing of *Otof ^−/−^* mice without the injection of AAVs or only injected with N-fragment AAVs was not improved (Fig. S5B). Compared to the *Otof ^−/−^* mice, the click ABR thresholds in the injected ear were restored to 58.80 ± 1.07 dB, which was about 20 dB higher the WT level (Fig. 5C), while the click ABR thresholds in the contralateral ear were restored to 77.28 ± 2.21 dB at 4 weeks (Fig. S5C). In addition, we analyzed the ABR wave I amplitude and latency of the injected ears in *Otof ^−/−^* mice. The amplitude was significantly lower than WT (click stimulus at 90 dB; WT, 3.28 ± 0.15 μV; injected ears, 0.17 ± 0.02 μV; *P* < 0.01) (Fig. 5D), while the latency was almost restored to WT level (at 90 dB; WT, 1.32 ± 0.02 ms; injected ears, 1.61 ± 0.02 ms; *P* < 0.01) (Fig. 5E).

We also investigated the long-term efficacy of our treatments. Eight weeks after injection of dual AAVs, the hearing of *Otof ^−/−^* mice was further improved by 5 to 10 dB in the injected ear (Fig. 5C and F), although the hearing in the contralateral ears was not further improved at all frequencies (Fig. S5D). At 16, 26, and 52 weeks after injection, the hearing of AAV-treated *Otof ^−/−^* mice were markedly improved compared with untreated *Otof ^−/−^* mice in the injected ear (Fig. 5G to I) and the contralateral ear (Fig. S5E and F). These results indicate that our therapeutic system can maintain its therapeutic effects for a relatively long time.

The hearing of adult mice with hereditary deafness is difficult to rescue [14,41], but for gene therapy of hereditary hearing loss, restoring the hearing of adult mice is more meaningful because the inner ear HCs of newborns are fully developed in humans [14]. Therefore, we injected the dual AAVs into the inner ear of *Otof ^−/−^* mice on P30. As shown in Fig. 5J, at 6 weeks after the injection, the hearing of *Otof ^−/−^* mice was restored by 20 to 40 dB in the injected ear. Excitingly, the restoration of auditory function was maintained for at least 14 weeks in adult *Otof ^−/−^* mice (Fig. 5K). These results suggest that the expression of a therapeutic protein driven by the mini-Myo15 promoter was able to treat adult mice with hereditary deafness.

### Potential use of the mini-Myo15 promoter in gene therapy of hereditary hearing loss

In order to further extend the application range of the mini-Myo15 promoter, we searched https://hereditaryhearingloss.org/ and PubMed for recessive nonsyndromic hearing loss genes that are expressed in inner ear HCs, and we identified the lengths of the CDS in these genes at the National Center for Biotechnology Information. Considering the ~4.7-kb cargo capacity of AAV, after accounting for the inverted terminal repeats, polyadenylation sequences, and the mini-Myo15 promoter, the transgenes could only possess a 0- to 3.4-kb or 3.4- to 6.8-kb CDS for single AAV or dual AAV vectors, respectively. As shown in Table S1, we identified 76 human recessive nonsyndromic hearing loss genes that are expressed in HCs in the inner ear, and 85.5% (65 out of 76) of these genes have a CDS shorter than 3.4 kb, which could be loaded by a single AAV, while 9.2% (7 out of 76) of these genes have a CDS longer than 3.4 kb but shorter than 6.8 kb, which could be delivered via dual AAVs (Table S1 and Fig. S6). This indicated that the length of the mini-Myo15 promoter makes it possible to facilitate AAV-mediated gene therapy for 94.7% of the HC-expressed genes that are related to recessive nonsyndromic hearing loss (Table S1 and Fig. S6).

## Discussion

In this study, we constructed a “multiple vectors in one AAV” strategy for promoter construction and engineering in vivo, and using this strategy, we truncated a 1,157-bp mid-Myo15 promoter and a 956-bp mini-Myo15 promoter that are specific to HCs. Both of these 2 promoters, especially the 1,157-bp mid-Myo15 promoter, were strong enough and specific enough to drive the expression of GFP and otoferlin in IHCs and to improve the auditory function of *Otof ^−/−^* mice. More importantly, under the control of the mini-Myo15 promoter, transgenes delivered by the AAV-PHP.eB-mini-Myo15-promoter-GFP were not expressed in the CNS, which reduced the toxicity of the transgenes. These results suggest that our promoter engineering strategy is effective.

The mini-Myo15 promoter restricts the expression of GFP delivered by AAV-PHP.eB to HCs. In order to test whether the mini-Myo15 promoter enables control over the expression of a functional molecule, we designed a dual-AAV approach via intein-mediated otoferlin protein recombination, and the expression of *OTOF* was driven by the mini-Myo15 promoter. These manipulations restored the hearing of *Otof ^−/−^* mice. We searched for recessive nonsyndromic hearing loss genes that are expressed in HCs, and we identified the length of the CDS of these genes. Based on these findings, we estimate that our 956-bp mini-Myo 15 promoter could be utilized to correct 94.7% (72 out of 76) of the genes that cause human recessive nonsyndromic hearing loss via single or dual-AAV-mediated gene therapy. Furthermore, single-AAV-delivered base editing systems have been developed to efficiently and precisely edit pathogenic single-nucleotide polymorphisms [48], and our mini-Myo15 promoter can also be used in gene therapy of dominant nonsyndromic hearing loss using such systems.

In addition to the 956-bp mini-Myo 15 promoter, we also engineered a 1,157-bp HC-specific mid-Myo15 promoter. This promoter is strong enough and specific enough to drive the expression of GFP in HCs. Although it has been reported that an overloaded AAV genome cannot be completely packaged and that the transduction efficiency of oversized AAVs is lower [49,50], in this study, we overloaded the 1,157-bp mid-Myo15 promoter to drive the expression of the *OTOF* in our gene therapy system and restored the expression of otoferlin in IHCs. At the same time, this manipulation restored the hearing of *Otof ^−/−^* mice to WT levels. These results demonstrate that the 1,157-bp mid-Myo15 promoter is a strong and HC-specific promoter that can be further used in gene therapy of hereditary hearing loss caused by other genes and for the clinical translation of *OTOF* gene therapy.

As viral vectors, AAVs are the most attractive delivery tools for gene therapy [11,51,52]. However, there are several limitations for the use of AAV-mediated gene therapy for hereditary deafness. The greatest limitation of recombinant AAVs is the low cargo capacity (~4.7 kb), which restricts the potential application of these viral vectors for larger genes with longer CDSs such as *OTOF* (6-kb-long cDNA), *MYO7A*, *STRC*, and *CDH23* [31,53]. In order to circumvent this problem, a popular strategy is to split large cDNAs sequences into 2 or more parts, with each AAV vector delivering part of the CDS, thus generating 2 or more AAV vectors. Once these split-AAV vectors transfect the same cells, full-length protein can be expressed in these cells. Presently, mRNA and intein trans-splicing strategies have been developed to deliver large transgenes [32,43]. Between these 2 strategies, intein-mediated protein splicing has been reported to perform at a relatively higher efficiency [44]. Therefore, in this study, we used an intein-mediated protein splicing strategy to deliver the 6-kb *OTOF* cDNA to rescue the hearing of *Otof ^−/−^* mice.

In addition to the low packaging capacity, another concern with AAVs is the transduction efficiency and the infection precision. The inner ear mainly consists of HCs, SCs, and spiral ganglion neurons, and a large number of genes are specifically expressed only in these cells [18,52]. In order to precisely and effectively rescue the deafness caused by mutations of genes expressed in these cells, cell-type specificity and high transduction efficacy are needed for AAV variants. Presently, 13 natural AAV serotypes and hundreds of AAV variants have been reported and engineered to transduce different type of cells [54]. Among these AAV variants, several serotypes have been applied in the gene therapy of inner ear disorders. Generally speaking, AAV1, AAV2/1, AAV5, AAV8, AAV9, AAV-PHP.eB, AAV2.7m8, AAV-ie, and AAV-ie-K558R can consistently transduce IHCs in mice, and some AAV variants have high transduction efficiency [34,35,55–57]; for example, AAV-PHP.eB, Anc80L65, AAV-ie, AAV2.7m8, and AAV-ie-K558R consistently transduce OHCs with a relatively high efficiency [34,35,55–57], while AAV.DJ, AAV-ie, AAV2.7m8, and AAV-ie-K558R transduce SCs with high efficiency [33–35,55–57] and AAV1 and AAV2/1 can transduce cells in spiral ganglion neurons [18,58]. In addition, several studies have demonstrated that AAV9-PHP.B and AAV-S efficiently transduce IHCs, OHCs, and SCs in nonhuman primates [59,60] and that AAV9-PHP.B can deliver GFP to human cochlear HCs and vestibular HCs [61]. While these findings show that some AAV variants transduce cochlear cells in an efficient manner, the cell-type specificity of these AAV variants need to be further improved. For example, in most cases, we cannot deliver the transgenes to OHCs via AAV vectors without also affecting IHCs.

Our previous results showed that dual-AAV-mediated *OTOF* gene therapy restored the hearing of *Otof ^−/−^* mice to WT levels. In that work, we used the ubiquitous CMV promoter to control the expression of otoferlin [41]. Proteins encoded by transgenes that are loaded by AAV can be harmful to untargeted cells [8], and in order to increase the safety of gene therapy, it is necessary to restrict the expression of transgenes in targeted cells using cell type-specific promoters [3,17,62]. Furthermore, engineered mini-promoters leave more space for transgenes in AAV-mediated gene therapy. About 6% of human protein-encoding genes have a CDS larger than 4 kb, and in order to fit the expression cassettes for the cDNA of these genes into a single AAV vector, less than 1 kb of space is left for the promoter and polyadenylation sequences. However, the majority of mammalian promoters are larger than 2 kb [19,63,64]. In the brain, the Pleiades Promoter Project has been implemented to develop small promoters to drive gene expression in the CNS in a highly brain region-specific manner [65], and this project has yielded a number of mini-promoters that target the cerebral cortex, hypothalamus, spinal cord, and retina [66,67]. At the same time, several other cell type-specific promoters have been developed to target different kinds of cells in CNS [11,12,19,68,69]. In the present study, a 1,157-bp mid-Myo15 promoter and a 956-bp mini-Myo15 promoter were engineered to facilitate gene therapy for HC-related recessive hereditary deafness. More importantly, our in vivo inner ear HC-specific promoter truncation strategy also provides suggestions for the construction of other cell type-targeted promoters. Taken together, our findings will promote the development of AAV-mediated gene therapy for hereditary deafness.

## Methods

### Animals

The *Otof ^−/−^* mice used in this study have been described previously [41]. C57BL/6J mice were used to inject AAVs loaded with GFP to evaluate the strength of the different promoters. Female and male mice were randomly chosen for all experiments. The mice were housed in groups of 6 in a ventilated and pathogen-free cage with 12-h dark/light cycles and free access to food and water. All handling of the animals and experimental protocols in this study were reviewed and approved by the Ethics Committee of Fudan University, China.

### The Myo15 promoter pool

The 1,611-bp Myo15 promoter consists of a 454-bp part 1 and a 1,157-bp part 2, and the 2 parts contain an 88- and 201-bp UTR, respectively. The 1,523-bp promoter 1 was generated by removing the 88-bp UTR in part 1 of the 1,611-bp Myo15 promoter; the 1,410-bp promoter 2 was generated by removing the 201-bp UTR in part 2 of the 1,611-bp Myo15 promoter; the 1,322-bp promoter 3 was generated by removing both the 88-bp UTR in part 1 and the 201-bp UTR in part 2 of the 1,611-bp Myo15 promoter; and the 1,157-bp promoter 4 was generated by removing the 454-bp part 1 of the 1,611-bp Myo15 promoter. We determined the necessity of the 454-bp part 1, the 88-bp UTR in part 1, and the 1,157-bp part 2 or 201-bp UTR of part 2 for maintaining the transcription initiation capacity of the 1,611-bp Myo15 promoter by comparing the function of these 4 promoters. We also deleted the phylogenetically unconserved DNA sequence by comparing and analyzing the sequences of the 1,157-bp part 2 with the predicted human and mouse Myo15 promoter, thus generating a 1,445-bp promoter 5 with part 1 and a 1,357-bp promoter 6 in which we deleted the 88-bp UTR of promoter 5. All 6 Myo15 promoter sequences were synthesized (Huajin), and each promoter was fused with the GFP sequence and the corresponding molecular barcode and then packaged together in AAV-PHP.eB. The expression levels of GFP driven by different promoters were measured, and the cochleae were harvested and the mRNA was isolated. The transcription initiation strength of each promoter compared to the others was determined via NGS and the unique molecular barcodes, and promoter strength was ranked based on enrichment of the molecular barcode in selected tissues. The top 5% to 10% performers were validated individually to identify which parts are necessary for the function of the 1,611-bp Myo15 promoter. After the 1,157-bp mid-Myo15 promoter was screened out, the mini-Myo15 promoter and the mini-Myo15-2 promoter were generated by removing the UTR of the mid-Myo15 promoter and by exchanging the UTR of the mid-myo15 promoter with an 88-bp UTR (the 1,611-bp Myo15 promoter contains a 201-bp and an 88-bp UTR, while the mid-Myo15 promoter contains only a 201-bp UTR), respectively. The 2 mini promoters were then packaged separately into recombinant AAV vectors to control the expression of GFP. All promoter sequences generated in this paper are provided in the Supplementary Materials.

### AAV plasmid construction

The synthesized Myo15-related promoter sequences were subcloned into pAAV-CMV-GFP, replacing the CMV promoters. For gene therapy in *Otof ^−/−^* mice, the human otoferlin CDS (NM_001287489.2) was separated into 2 fragments from site S2 as described previously [41], followed by packaging into 2 AAV-PHP.eB viruses (PackGene Biotech). The AAV vector plasmids included the mid-Myo15 promoter, the mini-Myo15 promoter, the Kozak sequence, and the poly-adenylation sequence. The vector plasmid together with capsid and helper plasmids was transiently transfected into human embryonic kidney 293T cells to produce viral particles. The particles were purified by iodixanol step-gradient centrifugation, dialyzed into phosphate-buffered saline (PBS), and subjected to quantitative PCR to confirm their VG titers. The VG titer of AAV-PHP.eB-GFP was 1 × 10^13^ VG/ml, while the VG titers of both AAV-PHP.eB-mini-Myo15-hOtof5-N-S2-Rma-N-intein and AAV-PHP.eB-mini-Myo15-Rma-C-intein-hOtof5-C-S2 were 2 × 10^13^ VG/ml.

### AAV administration

The virus was delivered to the right ear via the RMW as described previously [41]. Newborn mice were anesthetized by induced hypothermia followed by maintenance on an ice pack during the operation. For the adult mice, anesthesia was induced with ketamine (100 mg/kg) and xylazine (10 mg/kg) through intraperitoneal injection. A Nanoliter Microinjection System (WPI) connected to a glass micropipette (WPI, Sarasota, FL) was used to deliver the viruses (1 μl of AAV-PHP.eB-myo15-GFP or 1 μl of mixed therapeutic agent [vectors of N-terminal and C-terminal otoferlin mixed at 1:1 ratio by volume]). The speed of the injection was controlled at 5 nl/s for the newborn mice and 5 to 8 nl/s for the adult mice. After injection, standard postoperative care was applied to these mice.

### ABR measurement

ABR measurements were made at 37°C using a TDT BioSigRP system (Tucker-Davis Technologies, Alachua, FL, USA) in a soundproof chamber at different time points after injection as previously described [41]. Mice were anesthetized via intraperitoneal injection of ketamine (100 mg/kg) and xylazine (10 mg/kg), and the ABR signals were recorded by 3 needle electrodes inserted into the mastoid portion the (recording electrode), the subcutaneous tissues of the vertex (the reference electrode), and the rump (the ground electrode). ABR responses were elicited by 5-ms tone pips and subsequently amplified 10,000 times, pass-filtered with a 0.3- to 3-kHz passband, and averaged over 1,024 responses. Mice were presented tone burst stimuli of 4, 8, 16, 24, and 32 kHz at sound pressure levels between 20 and 90 dB in 5-dB steps until a threshold intensity that evoked a reproducible ABR waveform with an identifiable wave I peak was detected.

### Immunohistochemistry

In order to measure the strength of the different Myo15 promoters, cochlear whole mounts of injected and contralateral noninjected ears (P14 to P30) were immunostained as previously described with minor modifications [41] in order to test the transduction efficiency of the vector, the expression level of GFP, and the recombinant efficiency of human otoferlin in HCs in *Otof ^−/−^* mice. In order to evaluate the expression of GFP in the brain, brain slices were immunostained as previously described with minor modifications [9]. Freshly dissected cochleae were fixed in 4% fresh paraformaldehyde solution at room temperature for 1 h. Brains were excised and fixed in 4% fresh paraformaldehyde solution at 4°C for 48 h and sectioned at 50 μm with a vibratome/freezing microtome (Leica). The cochleae and brain slices were washed with PBS 3 times and decalcified in 10% EDTA for 1 to 2 d. The tissues were then permeabilized in 0.02% Triton X-100 for 30 min and blocked with PBS containing 1% Triton X-100 (1% PBST) and 10% donkey serum for 1 h at room temperature. The following primary antibodies were also diluted in 1% PBST and then incubated with the samples in Tris-buffered saline with Tween overnight at 4°C: chicken anti-GFP (ab13970, 1:500 dilution, Abcam) was used to measure the transduction efficiency of AAV-PHP.eB and the strength of the Myo15 promoters in the IHCs after injection of AAV-PHP.eB-CMV-GFP, AAV-PHP.eB-Myo15-GFP, AAV-PHP.eB-mid-Myo15-GFP, AAV-PHP.eB-mini-Myo15-GFP, AAV-PHP.eB-mini-Myo15-hOTOF-N-S2-Rma-N-intein, and AAV-PHP.eB-mini-Myo15-Rma-C-intein-hOTOF-C-S2, and anti-otoferlin (N terminus from mouse immunoglobulin G1 (IgG1), ab53233, 1:200, Abcam; C terminus from rabbit, PA552935, 1:200 dilution, Invitrogen) was used to test the recombinant efficiency of otoferlin in HCs after injecting the vectors. After rinsing 3 times in PBS, the samples were incubated at room temperature for 2 h with the following secondary antibodies: Alexa Fluor 555-conjugated anti-mouse IgG1 (A21127, 1:500 dilution, Invitrogen), Alexa Fluor 647-conjugated anti-rabbit IgG (A31573, 1:500 dilution; Invitrogen), or Alexa Fluor 488-conjugated anti-chicken IgY (ab63507, 1:500 dilution, Abcam). The samples were then counterstained with Fluoroshield with DAPI histology mounting medium (F6057, Sigma). Images were acquired with a confocal laser-scanning microscope (Zeiss, Oberkochen, Germany) with a 10× air objective (numerical aperture = 0.40) and a 63× oil-immersion objective (numerical aperture = 1.15). Maximum intensity projections of optical confocal sections were processed and analyzed by ZEN 2011 software (Zeiss) and ImageJ software (National Institutes of Health, http://imagej.net/).

### Statistical analysis

The raw enrichment of the molecular barcode in the inner ear was determined via NGS. The percentage of different molecular barcodes in the total barcode in the injected inner ear was counted. Then, barcode counts were normalized to generate within-sample relative abundances of the respective constructs. For each sample, the relative abundance of each promoter construct observed in the mRNA-derived cDNA library was divided by the relative abundance observed in the corresponding DNA library [70].

Statistical analysis was performed with Prism 7 (GraphPad Software, La Jolla, CA, USA). Unpaired 2-tailed Student *t* tests were performed to compare differences between 2 groups, while the comparison among multiple groups was analyzed by 1-way ANOVA with Tukey’s post hoc test or Dunn’s multiple comparisons test. Data are presented as mean values ± standard error of the mean in the text; ns, not significant (*P* > 0.05); *P < 0.05, **P < 0.01, and ***P < 0.001.

## Data Availability

All data needed to evaluate the conclusions in the paper are presented in the paper and the Supplementary Materials. Further information and requests for resources should be submitted to Y.S. (email: yilai_shu@fudan.edu.cn), G.L. (email: genglin.li@fdeent.org), and H.L. (email: hwli@shmu.edu.cn).

## Supplementary Materials

Supplementary 1 Figs. S1 to S6 Sequence of the promoters Table S1
