# Supplementary material for: Engineering of the AAV-Compatible Hair Cell-Specific Small-Size Myo15 Promoter for Gene Therapy in the Inner Ear
Source: Research (Wash D C). 2024 Apr 25;7:0341. doi: 10.34133/research.0341 (PMC11045262; doi:10.34133/research.0341)
Supplement: Supplementary 1 — Figs. S1 to S6 Sequence of the promoters Table S1 [file research.0341.f1.zip › Sequence of the promoters.pdf]

Sequence of the promoters

Promoter 1:

GGTCTCACCCAGCATTTTCACTTCTAATAAGTTCAAATGTGATACGGCACCTTTCTAAAAATTAGT
TTTCAGGGAAATAGGGTTCAAAACTGGTAGTGGTAGGGTCCATTCTCACGACCCCCAGGCCTGC
TAACCCTGACCAAGCTACCTATTACTTACCCTCCTCTTTCTCCTCCTCCTCTTTCTCCTTCTCCTGC
TTCCCCTCTTCCTTCTCCCTCCCTTCCTCTCCCTCCTCCCCCTCCTTGGCTGTGATCAGATCCAGA
GCCTGAATGAGCCTCCTGACCCCACACCCCCACTAGCATGGGCCTGCAAGTGCCCAGAAGTCC
CTCCTGCCTCCTAAACTGCCCAGCCGATCCATTAGCTCTTCCTTCTTCCCAGTGAAAGAAGCAGG
CACAGCCTGTCCCTCCCGTTCTACAGAAAGGAAGCTACAGCACAGGGAGGGCCAAAGGCCTTC
CTGGGACTAGACAGTTGATCAACAGCAGGACTGGAGAGCTGGGCTCCATTTTTGTTCCTTGGTG
CCCTGCCCCTCCCCATGACCTGCAGAGACATTCAGCCTGCCAGGCTTTATGAGGTGGGAGCTGG
GCTCTCCCTGATGTATTATTCAGCTCCCTGGAGTTGGCCAGCTCCTGTTACACTGGCCACAGCCC
TGGGCATCCGCTTCTCACTTCTAGTTTCCCCTCCAAGGTAATGTGGTGGGTCATGATCATTCTATC
CTGGCTTCAGGGACCTGACTCCACTTTGGGGCCATTCGAGGGGTCTAGGGTAGATGATGTCCCC
CTGTGGGGATTAATGTCCTGCTCTGTAAAACTGAGCTAGCTGAGATCCAGGAGGGCTTGGCCAG
AGACAGCAAGTTGTTGCCATGGTGACTTTAAAGCCAGGTTGCTGCCCCAGCACAGGCCTCCCA
GTCTACCCTCACTAGAAAACAACACCCAGGCACTTTCCACCACCTCTCAAAGGTGAAACCCAAG
GCTGGTCTAGAGAATGAATTATGGATCCTCGCTGTCCGTGCCACCCAGCTAGTCCCAGCGGCTC
AGACACTGAGGAGAGACTGTAGGTTCAGCTACAAGCAAAAAGACCTAGCTGGTCTCCAAGCAG
TGTCTCCAAGTCCCTGAACCTGTGACACCTGCCCCAGGCATCATCAGGCACAGAGGGCCACCC
TGCAGCTCAGCCTACTACTTGCTTTCCAGGCTGTTCCTAGTTCCCATGTCAGCTGCTTGTGCTTTC
CAGAGACAAAACAGGAATAATAGATGTCATTAAATATACATTGGGCCCCAGGCGGTCAATGTG
GCGTAAGGGGCCCTGGGTCCTTAAGCTTCTGCCACTGGCTCCGGCATTGCAGAGAGAAGAGAA
GGGGCGGCAGAGCTGAACCTTAGCCTTGCCTTCCTGGGTACCCTTCTGAGCCTCACTGTCTTCT
GTGAGATGGGCAAAGTGCGGGTGTGACTCCTTGGCAACGGTGTTACACCAGGGCAGGTAAAGT
TGTAGTTATTTGTGGGGTACACCAGGACTGTTAAAGGTGTAACTAT

Promoter 2:

GGTCTCACCCAGCATTTTCACTTCTAATAAGTTCAAATGTGATACGGCACCTTTCTAAAAATTAGT
TTTCAGGGAAATAGGGTTCAAAACTGGTAGTGGTAGGGTCCATTCTCACGACCCCCAGGCCTGC
TAACCCTGACCAAGCTACCTATTACTTACCCTCCTCTTTCTCCTCCTCCTCTTTCTCCTTCTCCTGC
TTCCCCTCTTCCTTCTCCCTCCCTTCCTCTCCCTCCTCCCCCTCCTTGGCTGTGATCAGATCCAGA
GCCTGAATGAGCCTCCTGACCCCACACCCCCACTAGCATGGGCCTGCAAGTGCCCAGAAGTCC
CTCCTGCCTCCTAAACTGCCCAGCCGATCCATTAGCTCTTCCTTCTTCCCAGTGAAAGAAGCAGG
CACAGCCTGTCCCTCCCGTTCTACAGAAAGGAAGCTACAGCACAGGGAGGGCCAAAGGCCTTC
CTGGGACTAGACAGTTGATCAACAGCAGGACTGGAGAGCTGGGCTCCATTTTTGTTCCTTGGTG
CCCTGCCCCTCCCCATGACCTGCAGAGACATTCAGCCTGCCAGGCTTTATGAGGTGGGAGCTGG
GCTCTCCCTGATGTATTATTCAGCTCCCTGGAGTTGGCCAGCTCCTGTTACACTGGCCACAGCCC
TGGGCATCCGCTTCTCACTTCTAGTTTCCCCTCCAAGGTAATGTGGTGGGTCATGATCATTCTATC
CTGGCTTCAGGGACCTGACTCCACTTTGGGGCCATTCGAGGGGTCTAGGGTAGATGATGTCCCC
CTGTGGGGATTAATGTCCTGCTCTGTAAAACTGAGCTAGCTGAGATCCAGGAGGGCTTGGCCAG
AGACAGCAAGTTGTTGCCATGGTGACTTTAAAGCCAGGTTGCTGCCCCAGCACAGGCCTCCCA
GTCTACCCTCACTAGAAAACAACACCCAGGCACTTTCCACCACCTCTCAAAGCTGCAGCTCAGC
CTACTACTTGCTTTCCAGGCTGTTCCTAGTTCCCATGTCAGCTGCTTGTGCTTTCCAGAGACAAA

ACAGGAATAATAGATGTCATTAAATATACATTGGGCCCCAGGCGGTCAATGTGGCAGCCTGAGC
CTCCTTTCCATCTCTGTGGAGGCAGACATAGGACCCCCAACAAACAGCATGCAGGTTGGGAGC
CAGCCACAGGACCCAGGTAAGGGGCCCTGGGTCCTTAAGCTTCTGCCACTGGCTCCGGCATTG
CAGAGAGAAGAGAAGGGGCGGCAGAGCTGAACCTTAGCCTTGCCTTCCTGGGTACCCTTCTGA
GCCTCACTGTCTTCTGTGAGATGGGCAAAGTGCGGGTGTGACTCCTTGGCAACGGTGTTACACC
AGGGCAGGTAAAGTTGTAGTTATTTGTGGGGTACACCAGGACTGTTAAAGGTGTAACTAT

Promoter 3:
GGTCTCACCCAGCATTTTCACTTCTAATAAGTTCAAATGTGATACGGCACCTTTCTAAAAATTAGT
TTTCAGGGAAATAGGGTTCAAAACTGGTAGTGGTAGGGTCCATTCTCACGACCCCCAGGCCTGC
TAACCCTGACCAAGCTACCTATTACTTACCCTCCTCTTTCTCCTCCTCCTCTTTCTCCTTCTCCTGC
TTCCCCTCTTCCTTCTCCCTCCCTTCCTCTCCCTCCTCCCCCTCCTTGGCTGTGATCAGATCCAGA
GCCTGAATGAGCCTCCTGACCCCACACCCCCACTAGCATGGGCCTGCAAGTGCCCAGAAGTCC
CTCCTGCCTCCTAAACTGCCCAGCCGATCCATTAGCTCTTCCTTCTTCCCAGTGAAAGAAGCAGG
CACAGCCTGTCCCTCCCGTTCTACAGAAAGGAAGCTACAGCACAGGGAGGGCCAAAGGCCTTC
CTGGGACTAGACAGTTGATCAACAGCAGGACTGGAGAGCTGGGCTCCATTTTTGTTCCTTGGTG
CCCTGCCCCTCCCCATGACCTGCAGAGACATTCAGCCTGCCAGGCTTTATGAGGTGGGAGCTGG
GCTCTCCCTGATGTATTATTCAGCTCCCTGGAGTTGGCCAGCTCCTGTTACACTGGCCACAGCCC
TGGGCATCCGCTTCTCACTTCTAGTTTCCCCTCCAAGGTAATGTGGTGGGTCATGATCATTCTATC
CTGGCTTCAGGGACCTGACTCCACTTTGGGGCCATTCGAGGGGTCTAGGGTAGATGATGTCCCC
CTGTGGGGATTAATGTCCTGCTCTGTAAAACTGAGCTAGCTGAGATCCAGGAGGGCTTGGCCAG
AGACAGCAAGTTGTTGCCATGGTGACTTTAAAGCCAGGTTGCTGCCCCAGCACAGGCCTCCCA
GTCTACCCTCACTAGAAAACAACACCCAGGCACTTTCCACCACCTCTCAAAGCTGCAGCTCAGC
CTACTACTTGCTTTCCAGGCTGTTCCTAGTTCCCATGTCAGCTGCTTGTGCTTTCCAGAGACAAA
ACAGGAATAATAGATGTCATTAAATATACATTGGGCCCCAGGCGGTCAATGTGGCGTAAGGGG
CCCTGGGTCCTTAAGCTTCTGCCACTGGCTCCGGCATTGCAGAGAGAAGAGAAGGGGCGGCAG
AGCTGAACCTTAGCCTTGCCTTCCTGGGTACCCTTCTGAGCCTCACTGTCTTCTGTGAGATGGGC
AAAGTGCGGGTGTGACTCCTTGGCAACGGTGTTACACCAGGGCAGGTAAAGTTGTAGTTATTTG
TGGGGTACACCAGGACTGTTAAAGGTGTAACTAT

Promoter 4(mid-Myo15-promoter):
GGTCTCACCCAGCATTTTCACTTCTAATAAGTTCAAATGTGATACGGCACCTTTCTAAAAATTAGT
TTTCAGGGAAATAGGGTTCAAAACTGGTAGTGGTAGGGTCCATTCTCACGACCCCCAGGCCTGC
TAACCCTGACCAAGCTACCTATTACTTACCCTCCTCTTTCTCCTCCTCCTCTTTCTCCTTCTCCTGC
TTCCCCTCTTCCTTCTCCCTCCCTTCCTCTCCCTCCTCCCCCTCCTTGGCTGTGATCAGATCCAGA
GCCTGAATGAGCCTCCTGACCCCACACCCCCACTAGCATGGGCCTGCAAGTGCCCAGAAGTCC
CTCCTGCCTCCTAAACTGCCCAGCCGATCCATTAGCTCTTCCTTCTTCCCAGTGAAAGAAGCAGG
CACAGCCTGTCCCTCCCGTTCTACAGAAAGGAAGCTACAGCACAGGGAGGGCCAAAGGCCTTC
CTGGGACTAGACAGTTGATCAACAGCAGGACTGGAGAGCTGGGCTCCATTTTTGTTCCTTGGTG
CCCTGCCCCTCCCCATGACCTGCAGAGACATTCAGCCTGCCAGGCTTTATGAGGTGGGAGCTGG
GCTCTCCCTGATGTATTATTCAGCTCCCTGGAGTTGGCCAGCTCCTGTTACACTGGCCACAGCCC
TGGGCATCCGCTTCTCACTTCTAGTTTCCCCTCCAAGGTAATGTGGTGGGTCATGATCATTCTATC
CTGGCTTCAGGGACCTGACTCCACTTTGGGGCCATTCGAGGGGTCTAGGGTAGATGATGTCCCC

CTGTGGGGATTAATGTCCTGCTCTGTAAAACTGAGCTAGCTGAGATCCAGGAGGGCTTGGCCAG
AGACAGCAAGTTGTTGCCATGGTGACTTTAAAGCCAGGTTGCTGCCCCAGCACAGGCCTCCCA
GTCTACCCTCACTAGAAAACAACACCCAGGCACTTTCCACCACCTCTCAAAGGTGAAACCCAAG
GCTGGTCTAGAGAATGAATTATGGATCCTCGCTGTCCGTGCCACCCAGCTAGTCCCAGCGGCTC
AGACACTGAGGAGAGACTGTAGGTTCAGCTACAAGCAAAAAGACCTAGCTGGTCTCCAAGCAG
TGTCTCCAAGTCCCTGAACCTGTGACACCTGCCCCAGGCATCATCAGGCACAGAGGGCCACC

Promoter 5:
CTGCAGCTCAGCCTACTACTTGCTTTCCAGGCTGTTCCTAGTTCCCATGTCAGCTGCTTGTGCTTT
CCAGAGACAAAACAGGAATAATAGATGTCATTAAATATACATTGGGCCCCAGGCGGTCAATGT
GGCAGCCTGAGCCTCCTTTCCATCTCTGTGGAGGCAGACATAGGACCCCCAACAAACAGCATG
CAGGTTGGGAGCCAGCCACAGGACCCAGGTAAGGGGCCCTGGGTCCTTAAGCTTCTGCCACTG
GCTCCGGCATTGCAGAGAGAAGAGAAGGGGCGGCAGAGCTGAACCTTAGCCTTGCCTTCCTGG
GTACCCTTCTGAGCCTCACTGTCTTCTGTGAGATGGGCAAAGTGCGGGTGTGACTCCTTGGCAA
CGGTGTTACACCAGGGCAGGTAAAGTTGTAGTTATTTGTGGGGTACACCAGGACTGTTAAAGGT
GTAACTATCATTTTCACTTCTAATAAGTTCAAATGTGATACGGCACCTTTCTAAAAATTAGTTTTC
AGGGAAATAGGGTTCAAAACTGGTAGTGGTAGGGTCCATTCTCACGACCCCCAGGCCTGCTAA
CCCTCCTATTACTTACCCTCCTCTTTCTCCTCCTCCTCTTTCTCCTTCTCCTGCTTCCCCTCTTCCTT
CTCCCTCCCTTCCTCTCCCTCCTCCCCCCTTGGCTGTGATCAGATCCAGAGCCTGAATGAGCCTC
CTGACCCCACACCCCCACTAGCATGGGCCTGCAAGTGCCCAGAATCCTAAACTGCCCAGCCTG
AAAGAAGCAGGCACAGCCTGTCCCTCCCGTTCTAAAGGAAGCTACAGCACAGGGAGGGCCAA
AGGCCTCCTGGGACTAGACAATCAACAGCAGGACTGGAGAGCTGGTTTGTTCCTTGGTGCCCTG
CCCCTCCCCATGACCTGCAGAGACATCAGCCTGCCAGGCTTTATGAGGTGGGAGCTGGGCTCTC
CCTGAGTTATTCAGCTCCCTGGAGTTGGCCAGCTCCTACACTGGCCACAGCCCTGGGCATCCGC
TTCTCACTTCTAGTTTCCCCTCCAAGGTAATGTGGTGGGTCATGATCATTCTATCCTGGCTTCAGG
GACCTGACTCCACTTTGGGGCCATTCGAGGGGTCTAGGGTAGATGATGTCCCCCTGTGGGGATT
AATGTCCTGCTCTGTAAAACTGAGCTAGCTGAGATCCAGGAGGGCTTGGCCAGAGACAGCAAG
TTGTTGCCATGGTGACTTTAAAGCCATGCTGCCCCGGCCTCCCAGTCTACCCTCACTAGAAAAC
AACACCCAGGCACTTTCCACCACCTCCCAAGGCTGGTCTAGAGAATGAATTATGGATCCTCGCT
GTCCGTGCCACCCAGACTGTAGGTTCAGTACAAGCAAAAAGACCTAGTCCCTGAACCTGTGAC
ACCTGCCCCAGGCATCATCAGGCACAGAGGGCCACC

Promoter 6:
CTGCAGCTCAGCCTACTACTTGCTTTCCAGGCTGTTCCTAGTTCCCATGTCAGCTGCTTGTGCTTT
CCAGAGACAAAACAGGAATAATAGATGTCATTAAATATACATTGGGCCCCAGGCGGTCAATGT
GGCGTAAGGGGCCCTGGGTCCTTAAGCTTCTGCCACTGGCTCCGGCATTGCAGAGAGAAGAGA
AGGGGCGGCAGAGCTGAACCTTAGCCTTGCCTTCCTGGGTACCCTTCTGAGCCTCACTGTCTTC
TGTGAGATGGGCAAAGTGCGGGTGTGACTCCTTGGCAACGGTGTTACACCAGGGCAGGTAAAG
TTGTAGTTATTTGTGGGGTACACCAGGACTGTTAAAGGTGTAACTATCATTTTCACTTCTAATAAG
TTCAAATGTGATACGGCACCTTTCTAAAAATTAGTTTTCAGGGAAATAGGGTTCAAAACTGGTAG
TGGTAGGGTCCATTCTCACGACCCCCAGGCCTGCTAACCCTCCTATTACTTACCCTCCTCTTTCTC
CTCCTCCTCTTTCTCCTTCTCCTGCTTCCCCTCTTCCTTCTCCCTCCCTTCCTCTCCCTCCTCCCCC

CTTGGCTGTGATCAGATCCAGAGCCTGAATGAGCCTCCTGACCCCACACCCCCACTAGCATGGG
CCTGCAAGTGCCCAGAATCCTAAACTGCCCAGCCTGAAAGAAGCAGGCACAGCCTGTCCCTCC
CGTTCTAAAGGAAGCTACAGCACAGGGAGGGCCAAAGGCCTCCTGGGACTAGACAATCAACA
GCAGGACTGGAGAGCTGGTTTGTTCCTTGGTGCCCTGCCCCTCCCCATGACCTGCAGAGACATC
AGCCTGCCAGGCTTTATGAGGTGGGAGCTGGGCTCTCCCTGAGTTATTCAGCTCCCTGGAGTTG
GCCAGCTCCTACACTGGCCACAGCCCTGGGCATCCGCTTCTCACTTCTAGTTTCCCCTCCAAGGT
AATGTGGTGGGTCATGATCATTCTATCCTGGCTTCAGGGACCTGACTCCACTTTGGGGCCATTCG
AGGGGTCTAGGGTAGATGATGTCCCCCTGTGGGGATTAATGTCCTGCTCTGTAAAACTGAGCTA
GCTGAGATCCAGGAGGGCTTGGCCAGAGACAGCAAGTTGTTGCCATGGTGACTTTAAAGCCAT
GCTGCCCCGGCCTCCCAGTCTACCCTCACTAGAAAACAACACCCAGGCACTTTCCACCACCTCC
CAAGGCTGGTCTAGAGAATGAATTATGGATCCTCGCTGTCCGTGCCACCCAGACTGTAGGTTCA
GTACAAGCAAAAAGACCTAGTCCCTGAACCTGTGACACCTGCCCCAGGCATCATCAGGCACAG
AGGGCCACC

Mini-Myo15-2-promoter:
GGTCTCACCCAGCATTTTCACTTCTAATAAGTTCAAATGTGATACGGCACCTTTCTAAAAATTAGT
TTTCAGGGAAATAGGGTTCAAAACTGGTAGTGGTAGGGTCCATTCTCACGACCCCCAGGCCTGC
TAACCCTGACCAAGCTACCTATTACTTACCCTCCTCTTTCTCCTCCTCCTCTTTCTCCTTCTCCTGC
TTCCCCTCTTCCTTCTCCCTCCCTTCCTCTCCCTCCTCCCCCTCCTTGGCTGTGATCAGATCCAGA
GCCTGAATGAGCCTCCTGACCCCACACCCCCACTAGCATGGGCCTGCAAGTGCCCAGAAGTCC
CTCCTGCCTCCTAAACTGCCCAGCCGATCCATTAGCTCTTCCTTCTTCCCAGTGAAAGAAGCAGG
CACAGCCTGTCCCTCCCGTTCTACAGAAAGGAAGCTACAGCACAGGGAGGGCCAAAGGCCTTC
CTGGGACTAGACAGTTGATCAACAGCAGGACTGGAGAGCTGGGCTCCATTTTTGTTCCTTGGTG
CCCTGCCCCTCCCCATGACCTGCAGAGACATTCAGCCTGCCAGGCTTTATGAGGTGGGAGCTGG
GCTCTCCCTGATGTATTATTCAGCTCCCTGGAGTTGGCCAGCTCCTGTTACACTGGCCACAGCCC
TGGGCATCCGCTTCTCACTTCTAGTTTCCCCTCCAAGGTAATGTGGTGGGTCATGATCATTCTATC
CTGGCTTCAGGGACCTGACTCCACTTTGGGGCCATTCGAGGGGTCTAGGGTAGATGATGTCCCC
CTGTGGGGATTAATGTCCTGCTCTGTAAAACTGAGCTAGCTGAGATCCAGGAGGGCTTGGCCAG
AGACAGCAAGTTGTTGCCATGGTGACTTTAAAGCCAGGTTGCTGCCCCAGCACAGGCCTCCCA
GTCTACCCTCACTAGAAAACAACACCCAGGCACTTTCCACCACCTCTCAAAG

Mini-Myo15-2-promoter:
GGTCTCACCCAGCATTTTCACTTCTAATAAGTTCAAATGTGATACGGCACCTTTCTAAAAATTAGT
TTTCAGGGAAATAGGGTTCAAAACTGGTAGTGGTAGGGTCCATTCTCACGACCCCCAGGCCTGC
TAACCCTGACCAAGCTACCTATTACTTACCCTCCTCTTTCTCCTCCTCCTCTTTCTCCTTCTCCTGC
TTCCCCTCTTCCTTCTCCCTCCCTTCCTCTCCCTCCTCCCCCTCCTTGGCTGTGATCAGATCCAGA
GCCTGAATGAGCCTCCTGACCCCACACCCCCACTAGCATGGGCCTGCAAGTGCCCAGAAGTCC
CTCCTGCCTCCTAAACTGCCCAGCCGATCCATTAGCTCTTCCTTCTTCCCAGTGAAAGAAGCAGG
CACAGCCTGTCCCTCCCGTTCTACAGAAAGGAAGCTACAGCACAGGGAGGGCCAAAGGCCTTC
CTGGGACTAGACAGTTGATCAACAGCAGGACTGGAGAGCTGGGCTCCATTTTTGTTCCTTGGTG
CCCTGCCCCTCCCCATGACCTGCAGAGACATTCAGCCTGCCAGGCTTTATGAGGTGGGAGCTGG
GCTCTCCCTGATGTATTATTCAGCTCCCTGGAGTTGGCCAGCTCCTGTTACACTGGCCACAGCCC

TGGGCATCCGCTTCTCACTTCTAGTTTCCCCTCCAAGGTAATGTGGTGGGTCATGATCATTCTATC
CTGGCTTCAGGGACCTGACTCCACTTTGGGGCCATTCGAGGGGTCTAGGGTAGATGATGTCCCC
CTGTGGGGATTAATGTCCTGCTCTGTAAAACTGAGCTAGCTGAGATCCAGGAGGGCTTGGCCAG
AGACAGCAAGTTGTTGCCATGGTGACTTTAAAGCCAGGTTGCTGCCCCAGCACAGGCCTCCCA
GTCTACCCTCACTAGAAAACAACACCCAGGCACTTTCCACCACCTCTCAAAGAGCCTGAGCCTC
CTTTCCATCTCTGTGGAGGCAGACATAGGACCCCCAACAAACAGCATGCAGGTTGGGAGCCAG
CCACAGGACCCAG
