# Supplementary material for: Engineering of the AAV-Compatible Hair Cell-Specific Small-Size Myo15 Promoter for Gene Therapy in the Inner Ear
Source: Research (Wash D C). 2024 Apr 25;7:0341. doi: 10.34133/research.0341 (PMC11045262; doi:10.34133/research.0341)
Supplement: Supplementary 1 — Figs. S1 to S6 Sequence of the promoters Table S1 [file research.0341.f1.zip › Table S1.pdf]

Table S1: Hair cell expressed deafness genes and their CDS length

Gene	CDS (bp)
FSCN2	1551
OTOF	5994
RDX	1752
POU4F3	1017
TRIOBP	1296
TPRN	2136
XIRP2	2817
ATOH1	1065
GFI1	1269
CDH23	1593
PCDH15	5868
DFNB59	1056
LHX3	1194
TMC1	2283
MYO15a	10593
MYO7a	6648
GRXCR1	873
PTPRQ	6900
LOXHD1	6636
CIB2	564
CABP2	663
EPS8	2469
EPS8L2	2148
ESPN	2565
STRC	5328
USH1C	2700
SLC26A5	1008
PIEZO2	8259
KCNA10	1536
CLRN1	699
CLRN2	699
GRXCR2	747
WHRN	2724
OCM	330
ISL1	1050
NTF3	774
TMTC4	2283
Ocm	330
Tbx2	2139
Ckb	1146
Slc7a14	2316
Lbh	318
TMIE	471
TMPRSS3	1365
GIPC3	939
OTOA	3420
CLDN14	720
CDC14A	1785
ILDR1	1509
ADCY1	3360
MARVELD2	1677
PDZD7	1554
PJVK	1059
DCDC2	1431
LHFPL5	660
S1PR2	1062
MSRB3	579
SYNE4	1215
GPSM2	2055
OTOGL	7062
TBC1D24	1662
ELMOD3	1176
KARS	1788
NARS2	1434
TSPEAR	2010
TMEM132E	3225
PPIP5K2	3669
CLIC5	756
FAM65B	3207
ROR1	2814
WBP2	786
ESRP1	1980
MPZL2	648

CEACAM16	1278
SPNS2	1650
CLDN9	654
