# Supplementary figures and images for: Engineering of the AAV-Compatible Hair Cell-Specific Small-Size Myo15 Promoter for Gene Therapy in the Inner Ear
Source: Research (Wash D C). 2024 Apr 25;7:0341. doi: 10.34133/research.0341 (PMC11045262; doi:10.34133/research.0341)

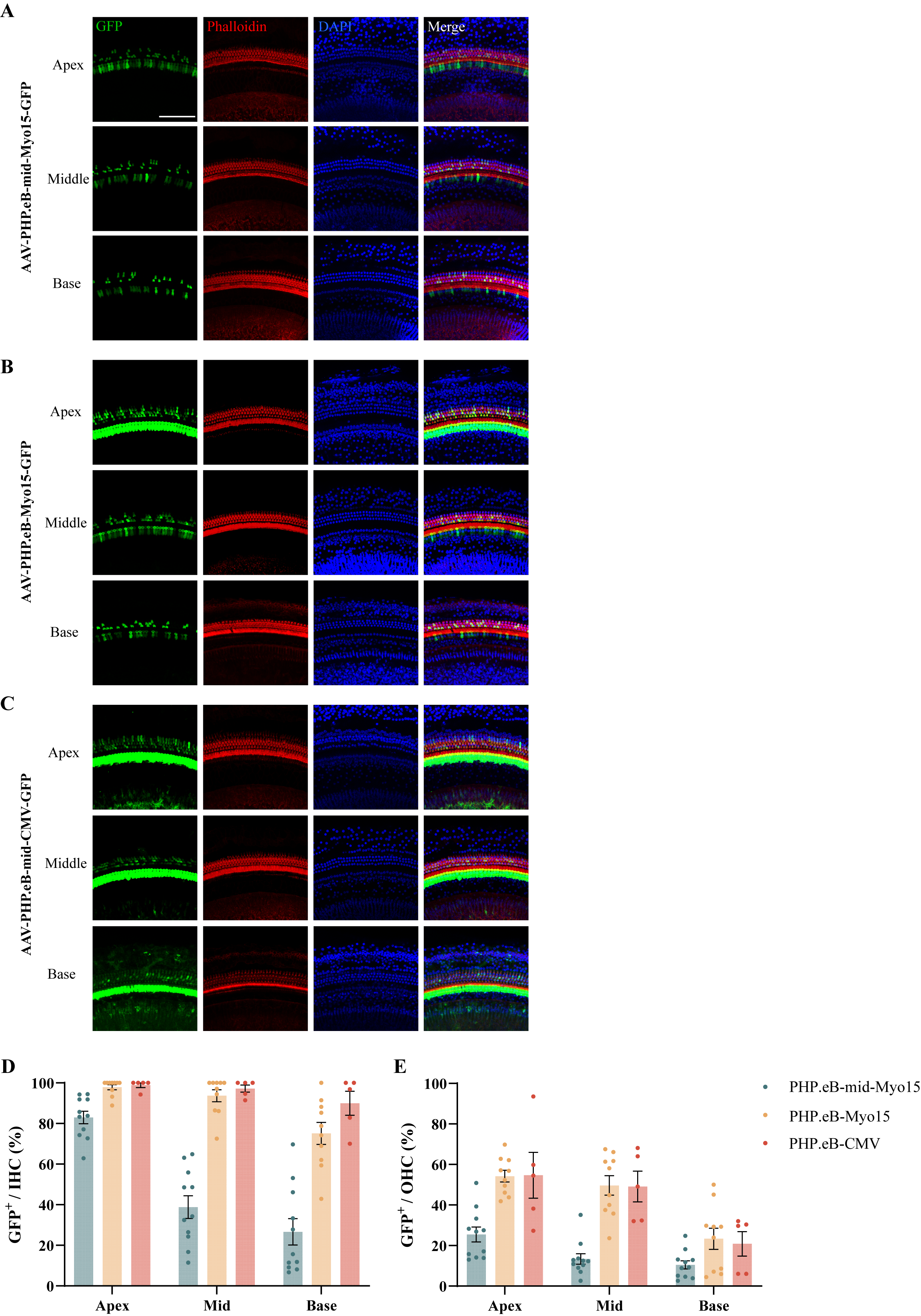

Supplement: Supplementary 1 — Figs. S1 to S6 Sequence of the promoters Table S1 [file research.0341.f1.zip › figure S1.tif]

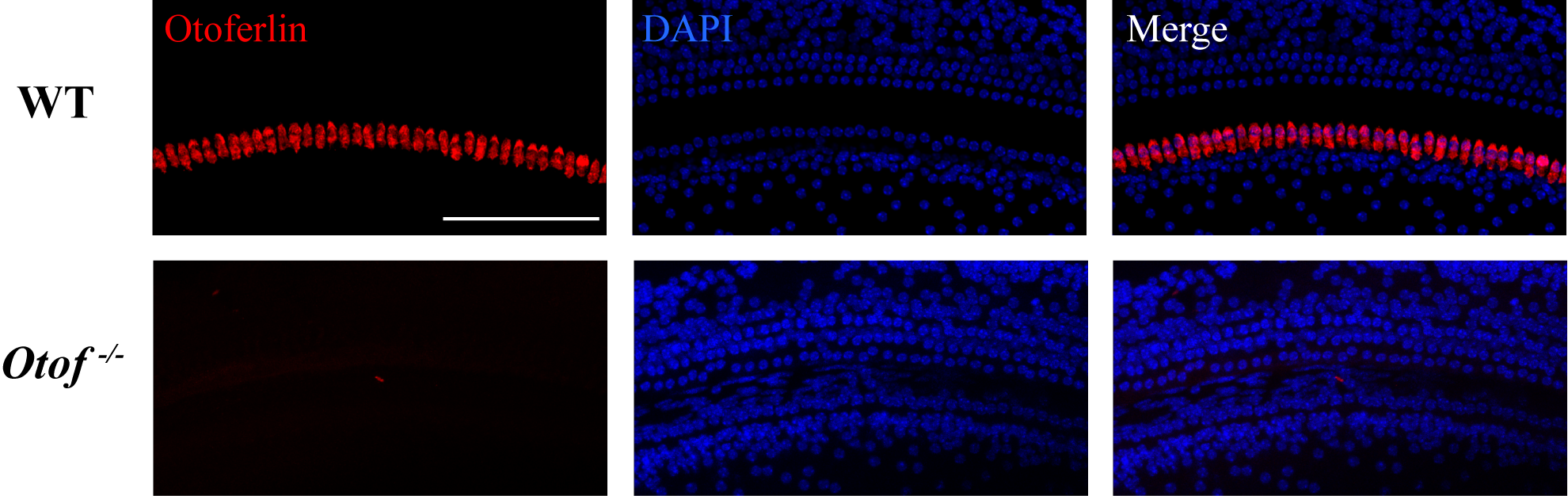

Supplement: Supplementary 1 — Figs. S1 to S6 Sequence of the promoters Table S1 [file research.0341.f1.zip › figure S2.tif]

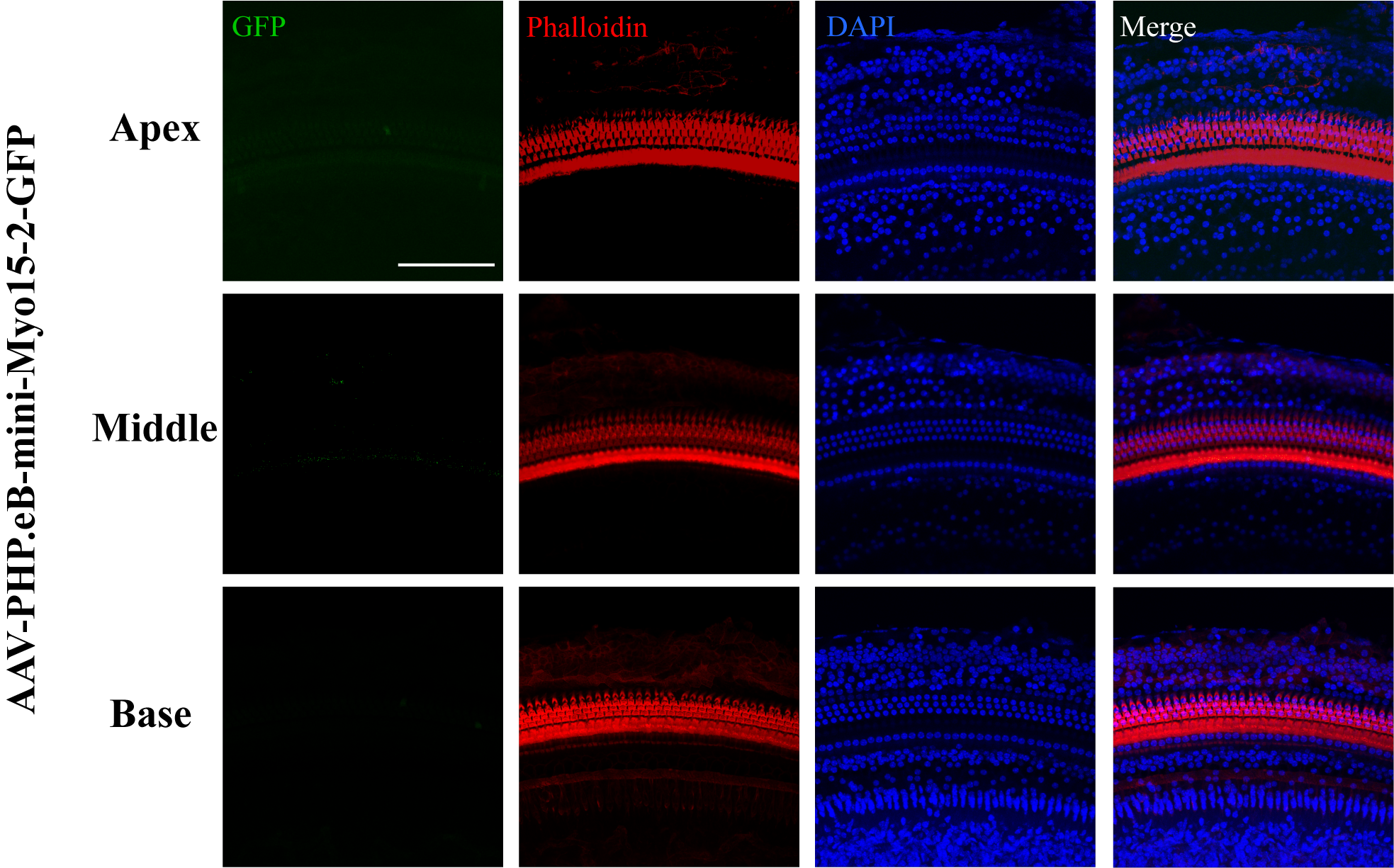

Supplement: Supplementary 1 — Figs. S1 to S6 Sequence of the promoters Table S1 [file research.0341.f1.zip › figure S3.tif]

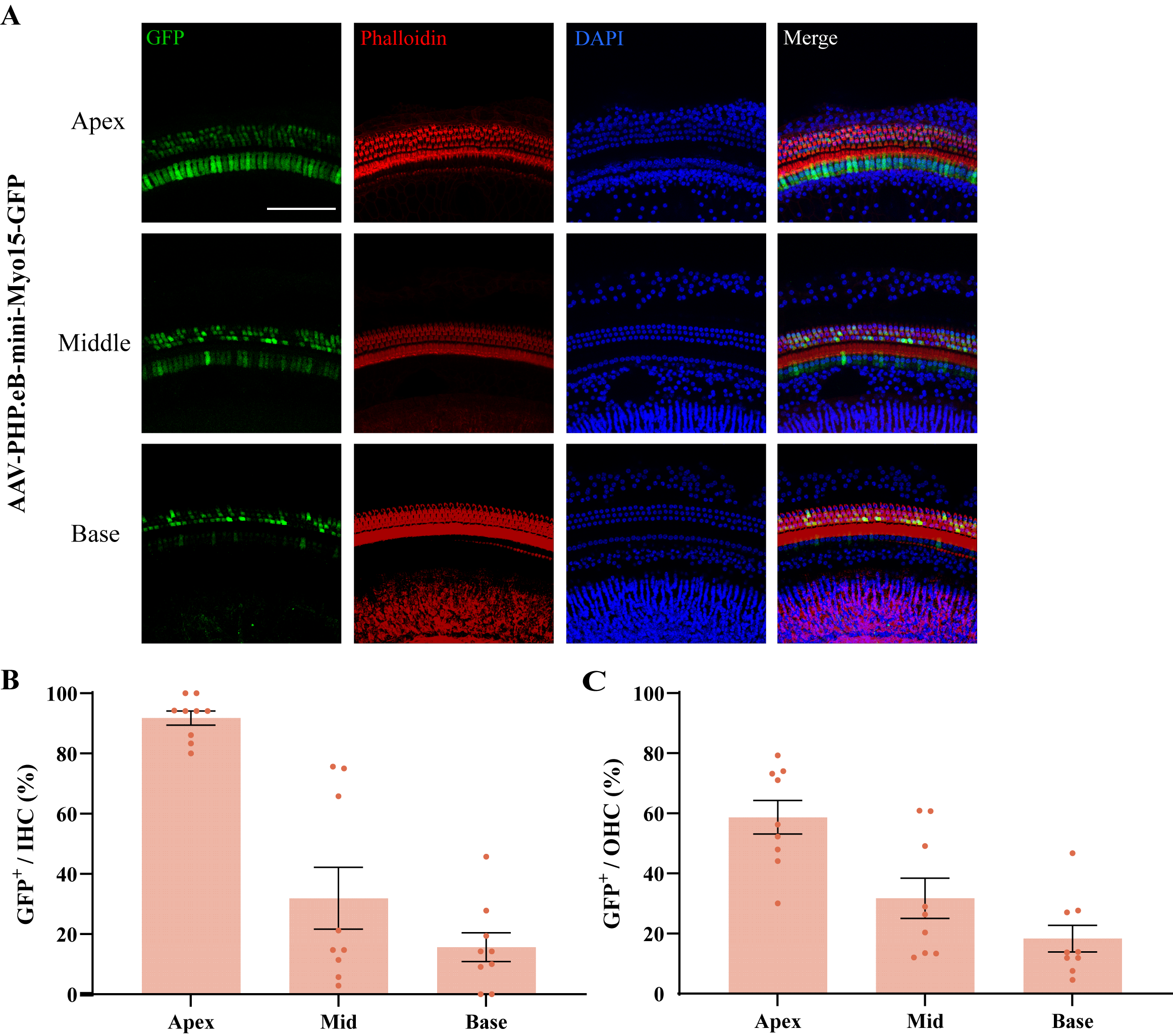

Supplement: Supplementary 1 — Figs. S1 to S6 Sequence of the promoters Table S1 [file research.0341.f1.zip › figure S4.tif]

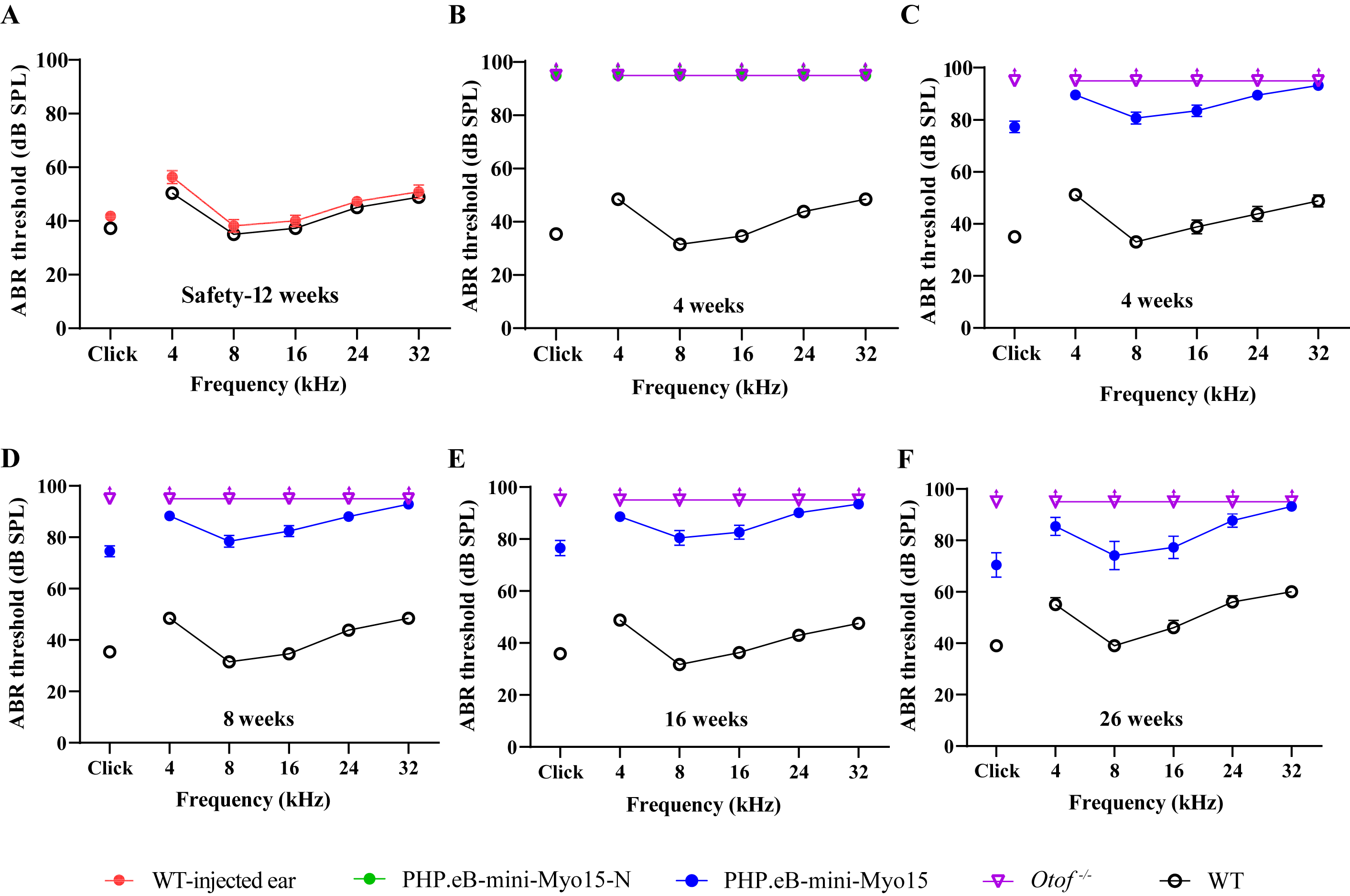

Supplement: Supplementary 1 — Figs. S1 to S6 Sequence of the promoters Table S1 [file research.0341.f1.zip › Figure S5.tif]

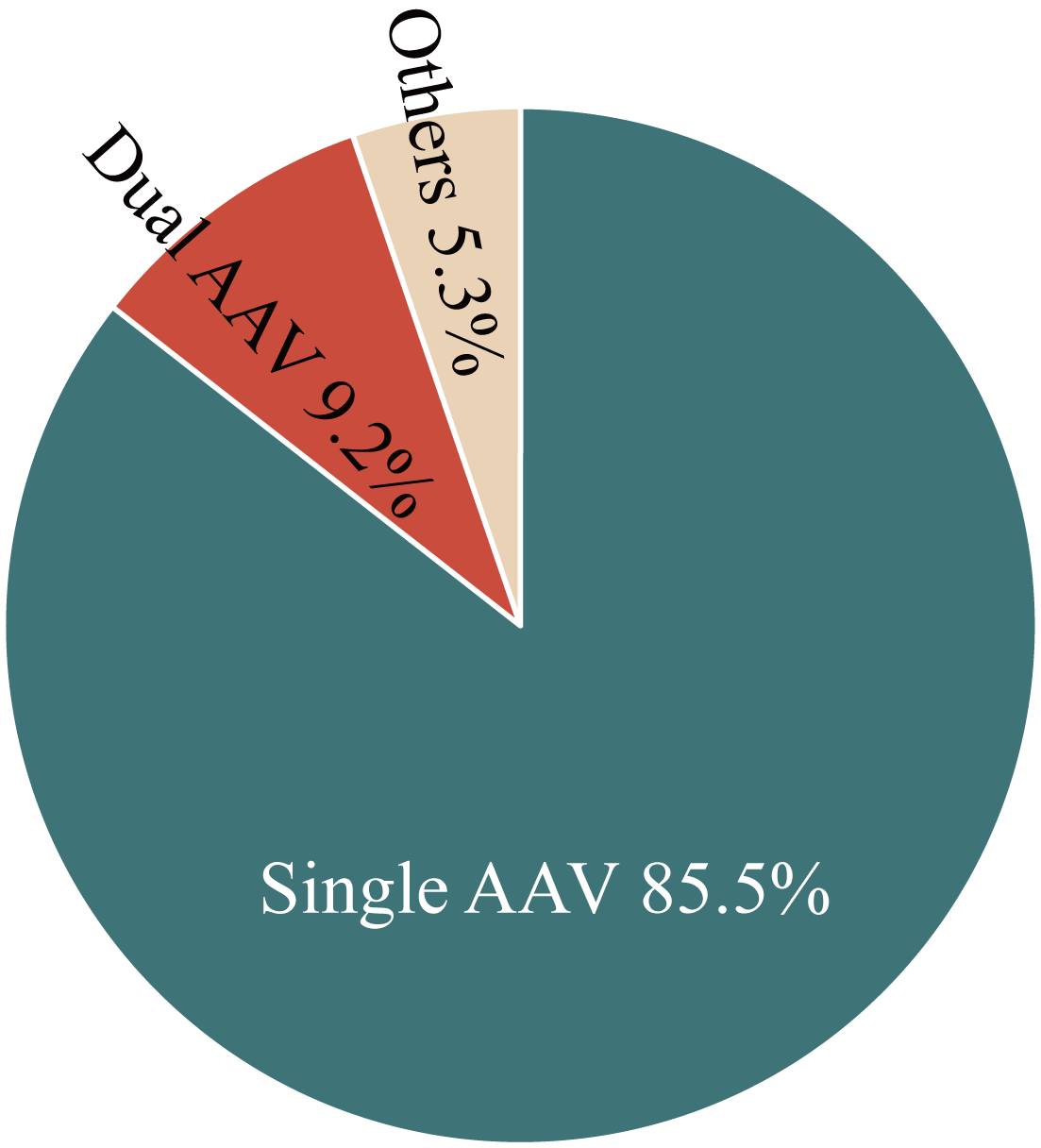

Supplement: Supplementary 1 — Figs. S1 to S6 Sequence of the promoters Table S1 [file research.0341.f1.zip › Figure S6.tif]
